# Iontophoresis‐Driven Microneedle Arrays Delivering Transgenic Outer Membrane Vesicles in Program that Stimulates Transcutaneous Vaccination for Cancer Immunotherapy

**DOI:** 10.1002/smsc.202300126

**Published:** 2023-10-02

**Authors:** Maoze Wang, Ge Yan, Qiyao Xiao, Nan Zhou, Hao-Ran Chen, Wei Xia, Lihua Peng

**Affiliations:** ^1^ College of Pharmaceutical Sciences Zhejiang University Hangzhou 310058 P. R. China; ^2^ Jinhua Institute of Zhejiang University Jinhua 321299 Zhejiang P. R. China; ^3^ State Key Laboratory of Quality Research in Chinese Medicine Macau University of Science and Technology Macau 999078 P. R. China; ^4^ Division of Applied Materials Science Department of Materials Science and Engineering Uppsala University Uppsala 751 05 Sweden

**Keywords:** cancer immunotherapy, iontophoresis, microneedle patches, transcutaneous vaccines, transdermal protein delivery, transgenic outer membrane vesicles

## Abstract

Transdermal delivery of antigen and chemokine proteins that activates the maturation of skin dendritic cells (DCs) and direct the migration of activated DCs to lymph and spleen is an important alternative to conventional vaccines. However, stratum corneum forms a barrier to skin penetration. The poor cellular uptake of free antigens and chemokines also limits transcutaneous immunization efficacy. In this work, a pair of iontophoresis‐driven microneedle patches is constructed, of which, two kinds of outer membrane vesicles (OMVs) derived from *Escherichia coli* transformed by plasmid encoding gp100 (IPMN‐G) and chemokine ligand 21 (IPMN‐C) are incorporated within microneedles, respectively. The topical application of IPMN‐G and IPMN‐C shows the effectiveness of transdermally delivering gp100 and CCL21 secreting vesicles to skin DCs. With iontophoresis as a driving generator, the release and uptake of transgenic OMVs in target cells are significantly enhanced, with transcutaneous immunization initiated. The in vivo applications of IPMN‐G and IPMN‐C with a 12 h interval retard the progression and prevent the occurrence of tumor spheroids. IPMN‐GC is shown as a promising triplatform in engineering transgenic OMV‐incorporated microneedles, driven by iontophoresis into a transcutaneous vaccine, providing a noninvasive system for the transdermal delivery of antigen and chemokine proteins for transcutaneous vaccination‐meditated immunotherapy.

## Graphical abstract

An IPMN‐GC‐based triplatform is constructed to provide a proof‐of‐concept strategy in engineering a bacterial‐derived outer membrane vesicles‐loaded porous microneedle patch driven by iontophoresis into a transcutaneous vaccine for the transdermal delivery of antigen gp100 and chemokine CCL21 in sequence. This platform has high delivery efficiency and great application potential in skin melanoma antitumor immunotherapy.

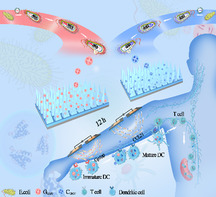

## 1 Introduction

Skin is the biggest organ with abundant antigen‐presenting cells (APCs) such as dendritic cells (DCs) including possessing great potential to initiate transcutaneous immunization for immunotherapy (TCI).^[^ 1, 2 ^]^ TCI mainly includes the activation of APCs and their migration to lymph nodes to boost the histocompatibility complex I (MHC I)‐restricted antigen presentation for robust cellular immunity.^[^ 3, 4 ^]^ The stimulation of transcutaneous immunization consequently needs to deliver not only antigen for DC activation but also chemokine to direct migration of DCs to lymph nodes. However, the transdermal delivery of both antigen and chemokine for transcutaneous immunization faces big challenges. First, the stratum corneum (SC) forms a strict barrier that tightly prevents the skin penetration of proteins.^[^ ^5^ ^]^ Second, the cellular uptake of proteins by APCs and migration of activated APCs to lymph nodes without stimulation will be too limited to induce sufficient MHC I‐restricted antigen presentation.^[^ 6, 7, 8 ^]^ Most current strategies only deliver antigens, sometimes with chemical adjuvants transferred together, to enhance immunization efficacy. Up to now, the programmed delivery of antigen and chemokine by a transdermal platform to initiate transcutaneous immunization has not been reported.

Meanwhile, antigen and chemokine proteins have a big molecular weight, high polarity, and fast degradation, which greatly limit their bioavailability and therapeutic efficacy.^[^ ^9^ ^]^ Despite that, polymeric and inorganic nanoparticles have been widely investigated as vehicles for protein delivery, the limited entrapment efficiency, relatively complicated fabrication and loading procedures, as well as the unwanted immune response induced is kept as an obstacle to be overcome.^[^ 10, 11, 12 ^]^ In the previous study,^[^ ^13^ ^]^ we constructed outer membrane vesicles (OMVs) derived from *Escherichia coli* transformed by plasmid DNAs (pDNAs) encoding target gene, showing the unique functions in secreting the targeting protein at the in situ site, making them promising nanoreservoirs and delivery vehicles for protein therapy.^[^ 14, 15 ^]^

In this study, to construct a simple, efficient, and safe transcutaneous vaccination platform, *E. coli* (T‐*E. coli*) transformed by pDNAs encoding gp100 and CCL21 genes, respectively, were prepared, from which, the OMVs secreting gp100 (G_OMV_) and CCL21 (C_OMV_) protein were isolated. G_OMV_ and C_OMV_ were then entrapped into a poly‐glycidyl methacrylate‐composed porous microneedle (MN) array with conductive properties (named MN‐G and MN‐C, respectively).

**Figure 1:**
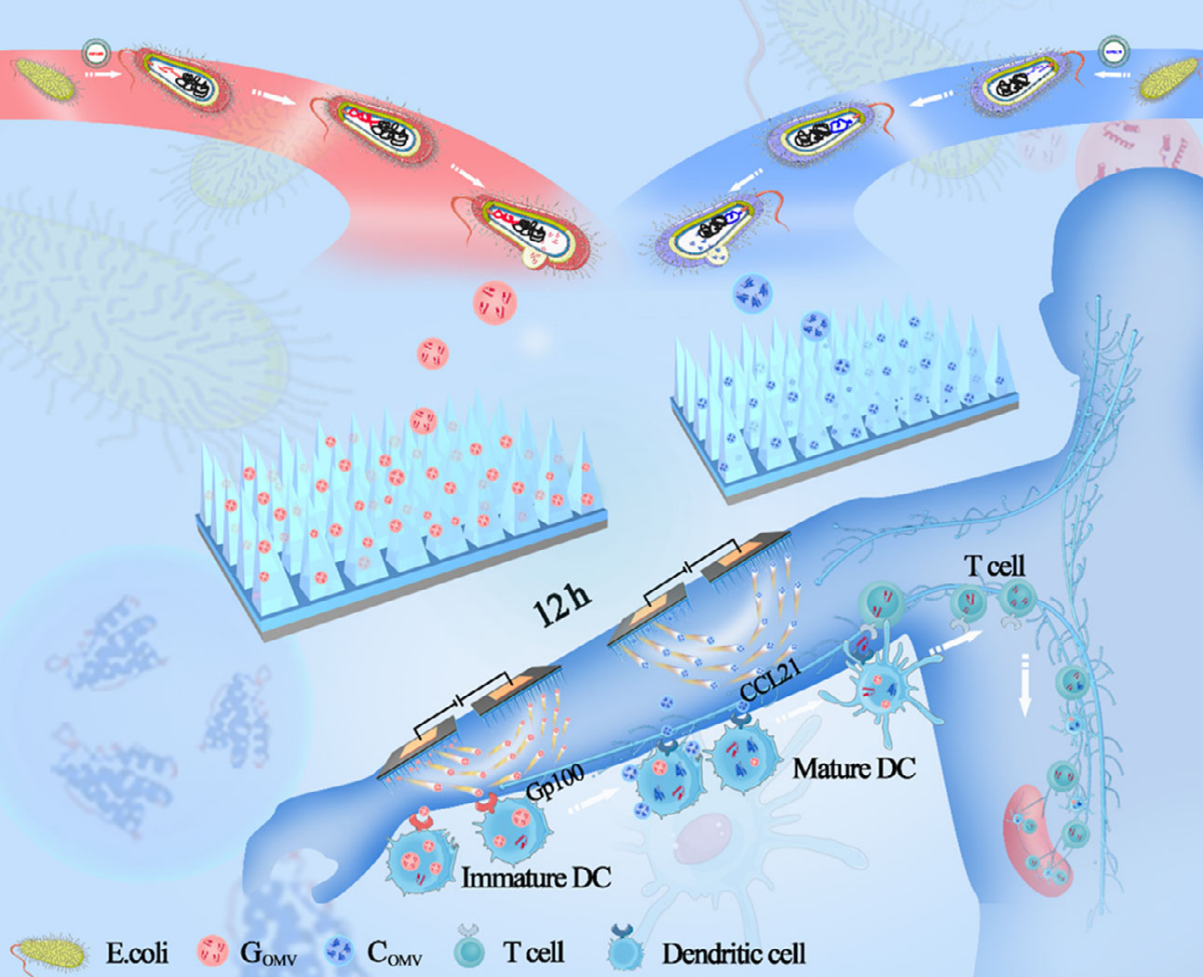
Schematic diagram of the action principle of IPMN‐GC. i) The target genes gp100 and CCL21 are transfected into *E. coli* by genetic engineering to extract OMV (G_OMV_ and C_OMV_) which carries the target protein. ii) Using MN to load G_OMV_ and C_OMV_ respectively to improve the transdermal efficiency and release of OMV. iii) The OMV is negatively charged, and the iontophoresis technology is used to promote the charged OMV to pass through the tissue barrier into the body through a low‐intensity DC electric field, thereby improving the transdermal penetration ability of the drug. v) The uptake of G_OMV_ by local skin DC cells can promote its maturation. After 12 h, the delivery of C_OMV_ enhances the migration of DC cells to lymph nodes through CCR7‐CCL21 after lymph node enrichment, thereby improving the antigen presentation of DCs to T cells and enhancing antitumor immune effects.

Iontophoresis (IP) that utilizes electronic current with physiological intensity^[^ 16, 17, 18 ^]^ is proposed as a power generator to stimulate the release of transgenic OMVs from MN patch and promote the flow of G_OMV_ and C_OMV_ to enhance the cellular uptake of G_OMV_ and C_OMV_ by target cells for enhanced bioavailability. The IP‐driven MN‐G (IPMN‐G) and MN‐C (IPMN‐C), forming a pair of patches (IPMN‐GC), are proposed to be topically applied to skin melanoma (for antitumor therapy) or normal body skin (for cancer vaccination) with a day‐and‐night model (12 h interval between the administration of IPMN‐G and IPMN‐C), to deliver the G_OMV_ and C_OMV_ through SC into the dermal layer in a program to activate the skin DCs and direct their migration to lymph nodes and spleen in sequence. IPMN‐GC is expected to be developed into an electronic–MN–exosome triplatform for the transdermal delivery of both antigen and chemokine proteins with high efficiency to induce vigorous transcutaneous immunization for cancer immunotherapy. The construction and working mechanism of IPMN‐GC are shown in **Figure** **1**.

## 2 Results

### 2.1 Preparation and Characterization of G_OMV_ and C_OMV_

**Figure 2:**
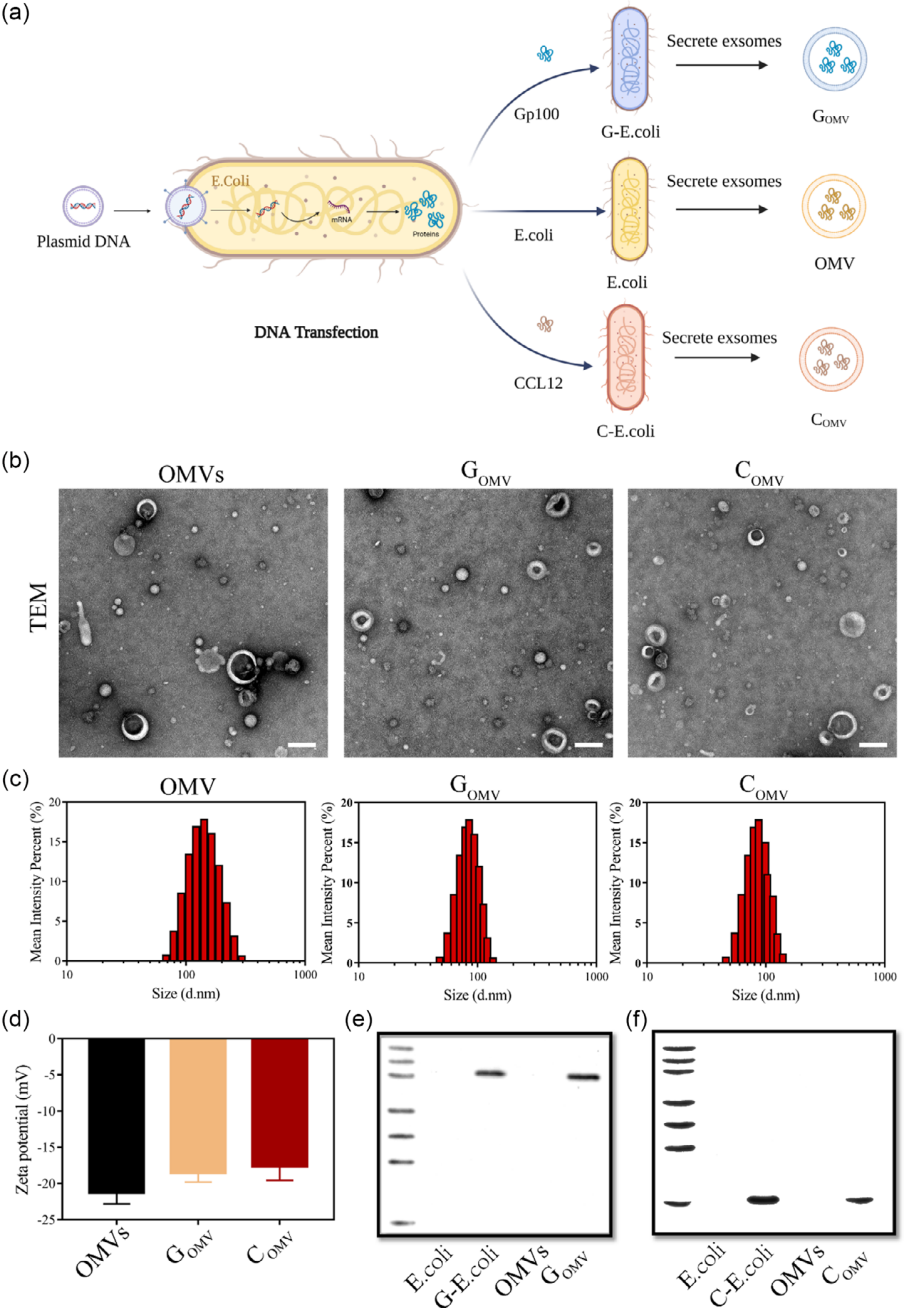
Construction and characterization of G_OMV_ and C_OMV_. a) Transformation of *E. coli* by pDNAs encoding target genes and the secretion of C_OMV_, G_OMV_, and OMVs. b) TEM images of OMV derived from ancestral *E. coli*, G_OMV_, and C_OMV_. Scale bar: 200 nm. c,d) The size distribution and zeta potentials of OMVs, G_OMV_, and C_OMV_. e,f) The identification of gp100 and CCL21 proteins in the tested samples.

*E. coli* was genetically engineered by transfected with pDNAs encoding human gp100 (gp100) or CCL21 to secret G_OMV_ and C_OMV,_ respectively (**Figure** **2**a). To remove the unwanted bacterial proteins, we screened and optimized the concentrations of the antibiotics to detoxify the prepared OMVs. Pretreatment of the OMVs by 20% antibiotics for 16–18 h was optimized as the final detoxification protocol. The detoxified OMVs not only has good biocompatibility with their treated cells even at the high concentration of 60 μg mL^−1^, keeping the viability of the treated cells over 80%, meanwhile, without any bacterial infection or obvious cytotoxicity observed (Figure S1, Supporting Information). Transformation of *E. coli* did not affect the particle size, zeta potential, and morphology of derived OMVs (Figure 2b). From Figure 2c, the average diameters of OMVs, G_OMV_ and C_OMV_ were around 93.59 ± 0.49, 98.12 ± 4.67, and 93.12 ± 3.14 nm, with the zeta potentials being −19.5 ± 0.45 and −18.1 ± 0.43 mV, respectively (Figure 2d). Gp100 protein was detected within G‐*E. coli* as well as its derived G_OMV_ (Figure 2e). No gp100 was found in the ancestral *E. coli* strain and its derived OMVs. The embedding of CCL21 protein in C_OMV_ is similar to G_OMV_ (Figure 2f). The concentrations of gp100 protein within G_OMV_ and CCL21 protein within C_OMV_ were determined as 425.2 ± 11.8 pg μg^−1^ OMVs and 513.4 ± 14.9 pg μg^−1^ OMVs, respectively.

### 2.2 Optimization and Characterization of IPMN‐GC

**Figure 3:**
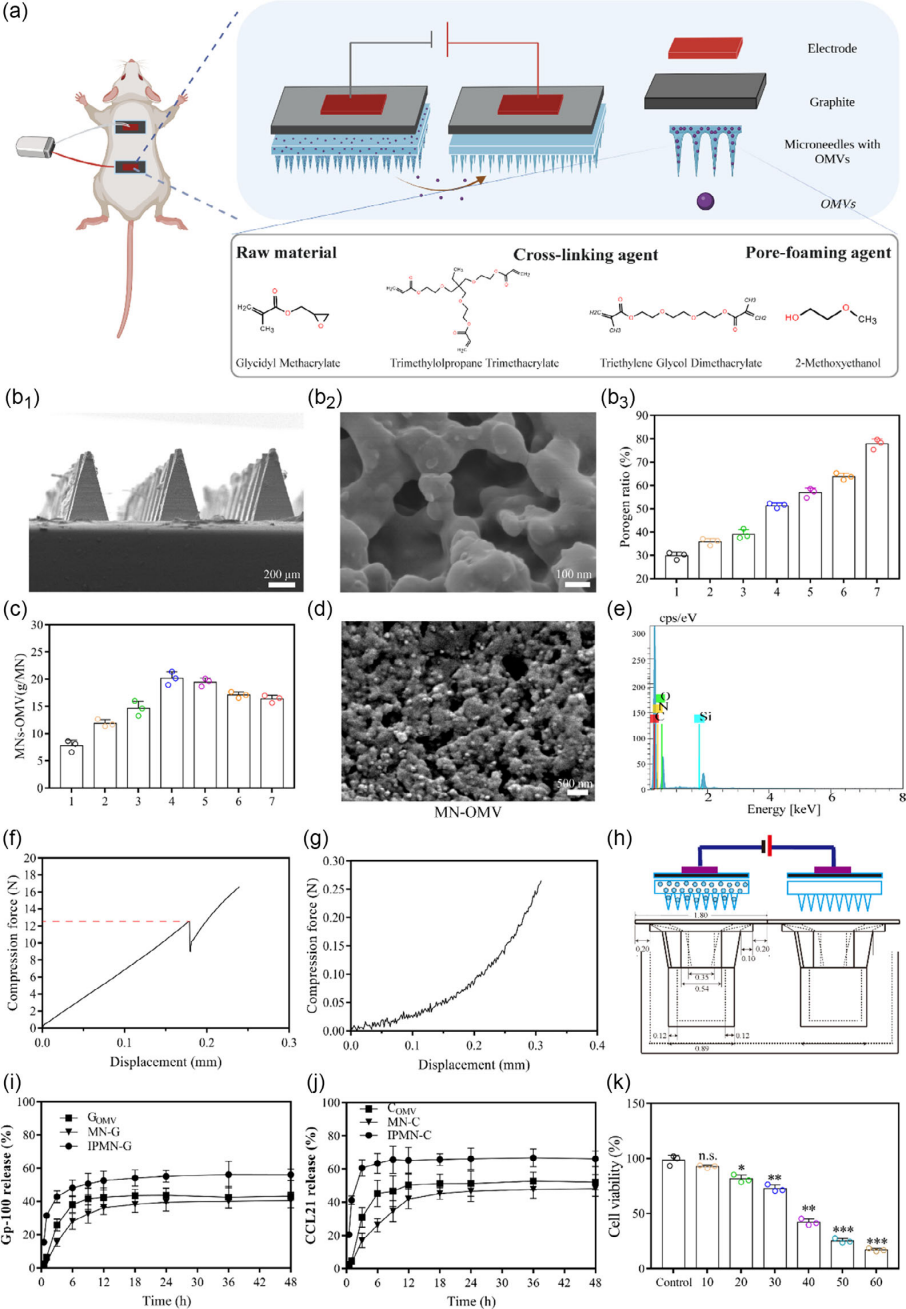
Fabrication and characterization of IPMN‐G and IPMN‐C. a) The composition and structure of MNs. b_1_) SEM images of MNs from different angles. b_2_) SEM image of MN surface. b_3_) Porosity of the MNs made from the two stock solutions of raw material mixed crosslinking agent and pore‐foaming agent in different ratios. c) Loading of OMVs in MNs with different porosity percentages. d) SEM image of MN‐OMV. e) EDS analysis of MN‐OMV. f,g) Characterization of the mechanical strength of MN samples: f) compressive fracture force of MNs; g) compression force versus displacement of MNs penetrating skin corneum. h) Schematic of the 3D scaffold model for in vitro tests. i) gp100 release profiles without or with IP (*n* = 5). j) CCL21 release profiles without or with IP (*n* = 5). k), Cell viability of BMDCs treated with IPMN under different electric intensities (voltage: 10, 20, 30, 40, 50, 60 V; working time: 30 s). (*p < 0.05, **p < 0.01, ***p < 0.001).

The composition and structure of MNs are shown in **Figure** **3**a. Blank porous MNs made of poly‐glycidyl methacrylate were prepared. G_OMV_ and C_OMV_ were then loaded into MNs, followed by the coating with graphite plate, forming the MN‐G_OMV_ and MN‐C_OMV_ with conductive properties. DC voltage regulator provides optional intensity voltage through the platinum‐plate electrode to form IPMN‐G and IPMN‐C. The negatively charged OMV migrated in electric field and implemented continuous transmission between PMNs, which is conducive to rapid local enrichment of OMVs for improving bioavailability.

As shown in Figure 3b_1_,b_2_ and S2, Supporting Information, the MN array composed of 12 × 12 needles is distributed on a 1 cm^2^ supporting matrix by a micromolding process. The as‐prepared MNs present pyramidal needle morphology. The total height of MNs and the tip‐to‐tip distance of adjacent MNs is about 630 and 200 μm, respectively. The supporting matrix has a diameter of about 300 μm. The average diameter of fixed pores is around 300 nm. To optimize the loading capacity of OMVs in MNs, MNs with different porosity rates were prepared and screened. From Figure 3b_3_, when the volume ratios of the PGMA to porogen used in the MN are 2:1, 1.72:1, 1.50:1, 1:1, 0.75:1, 0.60:1, 0.30:1, the corresponding porosities involved in the MN are 31.24%, 35.25%, 38.32%, 50.23%, 57.34%, 63.65%, 78.25%, respectively. Among them, the drug loading efficacy was increased with the increase of porosity and reached the maximum drug loading amount of 20 μg/array with a porosity of 50.23%. However, when the porosity was further increased, the drug loading decreased which might be partly contributed to the leakage of OMVs from the MNs with a high density of pores (Figure 3c). The MN formulation with this ratio (PGMA to porogen of 1:1) was therefore selected to be used in the subsequent investigations. By energy‐dispersive spectrometer analysis, OMVs inside the MNs were identified and demonstrated (Figure 3d,e and S3, Supporting Information).

Mechanical strength of MNs is one of the important parameters indicating the skin penetration capacity of MN. As shown in Figure 3f, as the loading displacement increased, the MN array exhibited a failure load of 12.56 N per needle. Figure 3g shows the smooth, continuous force–displacement curves of PMNs to puncture excised mouse skin. No fracture occurred when the force reached 0.25 N, which far exceeded 0.058 N, the minimum force required to penetrate human skin.^[^ ^19^ ^]^

A special 3D scaffold was fabricated to brace MNs or provide a cell growth platform for further in vitro investigation. The release of OMVs from MNs and their diffusion to the surrounding microenvironment are the preconditions of OMVs to provide therapeutic effects. Briefly, MN‐G_OMV_ and blank MN were put on two 3D scaffolds, respectively. Then direct current voltage was applied and the solution in chamber was collected for dialysis (Figure 3h). The release of gp100 or CCL21 from IPMN‐G or IPMN‐C was evaluated. From Figure 3i,j, only around 36% of gp100 and 41% of CCL21 were released from G_OMV_ and C_OMV_ groups, respectively within 48 h, when the addition of MNs even controlled the process. However, upon IP excitation, 56.13% of gp100 and 66.14% of CCL21 were released within 12 h, indicating the significant enhancement of iontophoresis in promoting the release of proteins from MNs‐OMVs. Current intensity affects not only the delivery efficiency but also biocompatibility of ionophoresis. It was shown in Figure 3k, the cell viability treated with IPMN at 10 V for 30 s was 91.45 ± 10.13%, indicating that mild current induced almost no toxicity to cells. IPMN penetrates SC into the skin epidermis and dermis with lower electrical resistance,^[^ ^20^ ^]^ thereby effectively reducing the applied voltage and improving in vivo safety.

**Figure 4:**
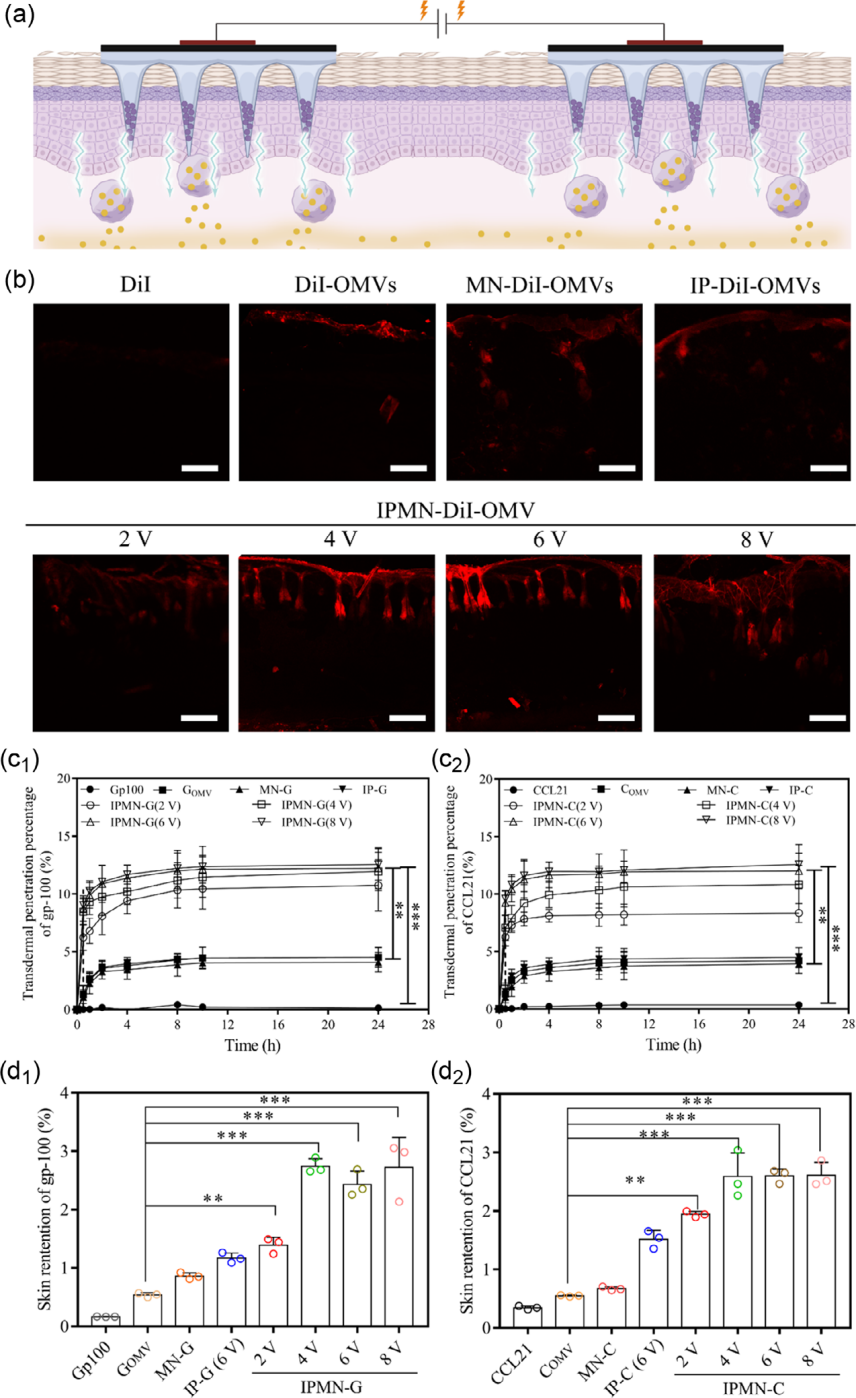
Transdermal delivery of gp100 and CCL21 proteins by IPMN‐G and IPMN‐C in vitro. a) Schematic representation of the facilitation in transdermal delivery of gp100 and CCL21 by IPMN‐G or IPMN‐C. b) CLSM images of skin treated by the tested samples for 30 min through topical application. Scale bar; 200 μm. c) Quantification of the amounts of permeated gp100 protein (c_1_) and CCL21 protein (c_2_) in vitro (*n* = 3). d) Quantification of the amounts of gp100 (d_1_) and CCL21 proteins (d_2_) in skins in vitro (*n* = 3). (*p < 0.05, **p < 0.01, ***p < 0.001).

Considering the transdermal delivery of OMVs for uptake by skin DCs as the critical step to initiate transcutaneous vaccination effects (**Figure** **4**a), we investigated the skin penetration efficacy of OMVs delivered by IPMN system. As shown in Figure 4b and S4, Supporting Information, the fluorescence of Dil‐OMVs was identified 200–300 μm deep in the skin, indicating that OMVs had the intrinsic transdermal ability. However, using IPMN as vehicle, the skin penetration of OMVs was enhanced significantly and can penetrate depth of 800 μm under IP stimulation at voltages of 6–8 V. The transdermal efficiency of gp100 and CCL21 proteins delivered by IPMN‐G and IPMN‐C under different voltage conditions was investigated. As shown in Figure 4c,d, compared with the proteins delivered by MN‐G and MN‐C with IP stimulation, of which, only 3.8 % of gp100 and 3.2% of CCL21 penetrated through skin within 24 h. In contrast, the IP stimulation at voltages of 2, 4, 6, and 8 V groups enhanced the cumulative skin penetration of gp100 3.2‐, 1.6‐, 1.5‐, and 1.9‐folds, respectively. A similar enhancement for IPMN‐C than MN‐C was also obtained (Figure 4c_1_). For example, when the applied voltage was 6 V, the cumulative transdermal amount of CCL27 increased 3‐folds by IPMN‐C than MN‐C (Figure 4c_2_). The retention of gp100 and CCL21 proteins in skin was also investigated and shown in Figure 4d_1_,d_2_. It can be seen that, compared with the MN‐G_OMV_ and MN‐C_OMV_ groups, the skin retention of G_OMV_ or C_OMV_ delivered by IPMN‐G or IPMN‐C group increased from 0.25 ± 0.09% to 2.66 ± 0.72% and 0.55 ± 0.15% to 2.54 ± 0.38%, respectively. These results give evidence that the presence of MN disrupts the skin barrier and help increase OMV SCs’ penetration. Once combined with IP, the transdermal efficacy of charged OMV was further enhanced by electric field. Consequently, the combination of MN and iontophoresis can act as efficient vehicles for the transdermal delivery of proteins with synergistic enhancement.

### 2.3 Cellular Uptake and Distribution of OMVs in BMDCs Delivered by IPMN‐G and IPMN‐C

**Figure 5:**
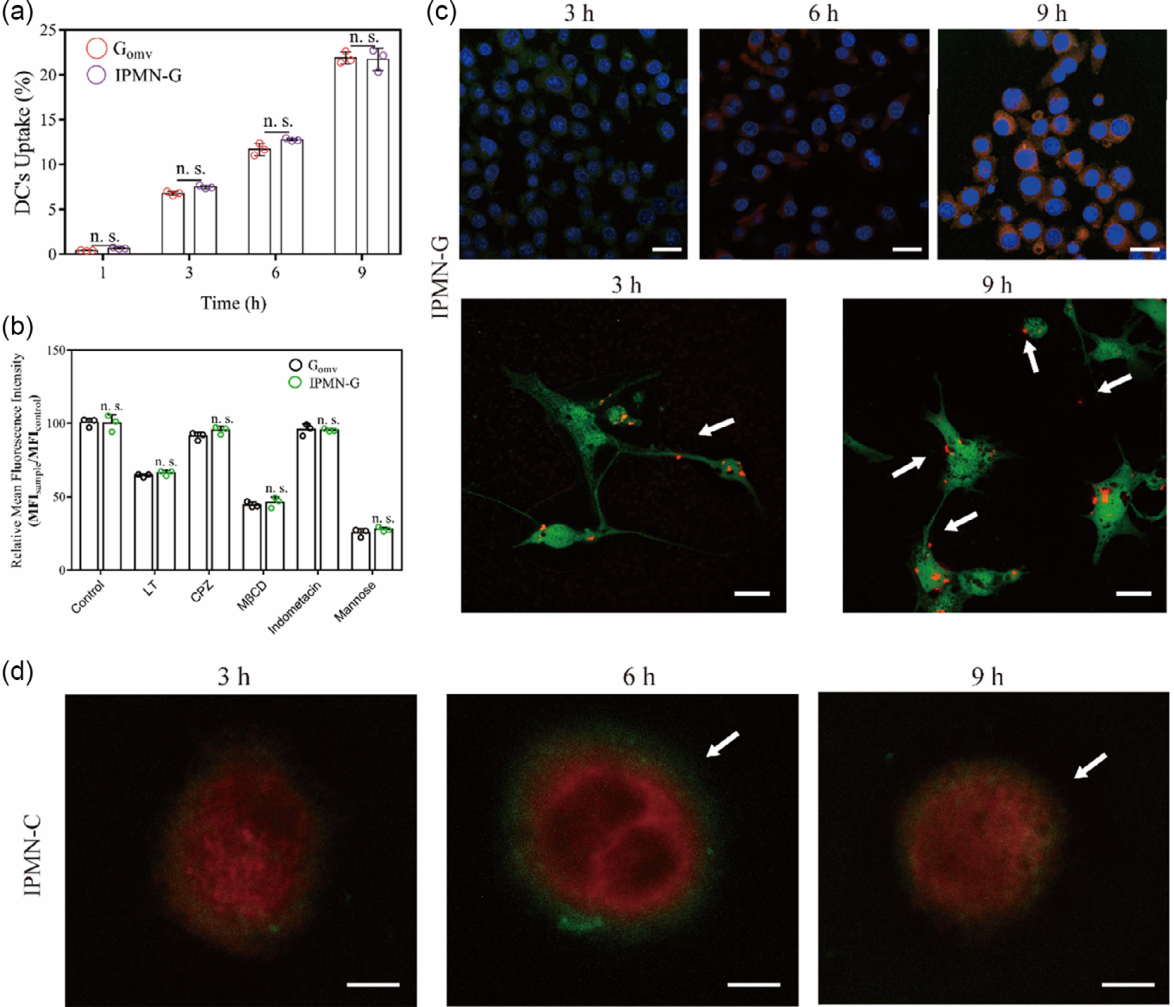
Cellular uptake and distribution of G_OMV_ delivered by IPMN in BMDCs. a,b) Cellular uptake (a) and intracellular pathways (b) of IPMN‐G delivered G_OMV_ in BMDCs (*n* = 3). c) The intracellular distribution of G_OMV_ and IPMN released G_OMV_ in BMDCs after treatment for 3, 6, and 9 h observed by CLSM; scale bar: 10 μm. Lysosome (green), G_OMV_ (red). d) The distribution of IPMN‐C for 3, 6, and 9 h observed by CLSM; scale bar: 5 μm.

To clarify the delivery mechanism of IPMN‐G and IPMN‐C, the cellular uptake of G_OMV_ and C_OMV_ by target cells was studied. The flow cytometry showed that G_OMV_ released from IPMN‐G can be taken up by bone marrow‐derived DCs (BMDCs) in a time‐dependent manner (**Figure** **5**a) and the uptake percentages reached 7.2%, 12.4%, and 22.7% at 3, 6, and 9 h, respectively. The mechanism of IPMN facilitation in the intracellular translocation of OMVs was studied as well. As shown in Figure 5b, the cellular uptake of G_OMV_ in IPMN‐G group in the low‐temperature, M‐βCD, and mannose pretreated groups was significantly decreased, which indicated that the intracellular mechanisms of G_OMV_ in DCs, delivered by IPMN involve the energy‐dependent, cholesterol‐dependent pathway, and the mannose‐mediated endocytosis.

The intracellular distribution of G_OMV_ in BMDCs was also observed by confocal laser scanning microscopy (CLSM). From Figure 5c and S5a, Supporting Information, a large amount of red fluorescence around the nucleus was observed at 9 h, suggesting that the G_OMV_ delivered by IPMN were ingested by BMDCs. The enlarged image showed the red partially separated away from the green fluorescence signal, indicating G_OMV_ can escape from the lysosome to deliver gp100 into the cytoplasm for inducing cellular immune responses. The binding of CCL21 to the receptor on membrane of BMDC is the precondition to its activation to BMDC. In Figure 5d and S5b, Supporting Information, C_OMV_ was shown to be enriched on the cell membrane of BMDC in IPMN‐C group; by contrast, less C_OMV_ were observed on the surface of BMDCs for C_OMV_ group. These results demonstrated that IPMN significantly enhanced the binding of CCL21 to the chemokine receptor 7 (CCR7) on the membrane of BMDCs, which might be contributed to the cell migration. These results indicated that the stimulation of IPMN in the cellular uptake of OMVs that might contribute to the iontophoresis‐induced electrorepulsion and electro‐osmosis can stimulate the uptake, endosome escape, and redistribution of OMVs inside cells.^[^ ^21^ ^]^

### 2.4 Influence of IPMN‐GC Treatment on the Behaviors of BMDCs

**Figure 6:**
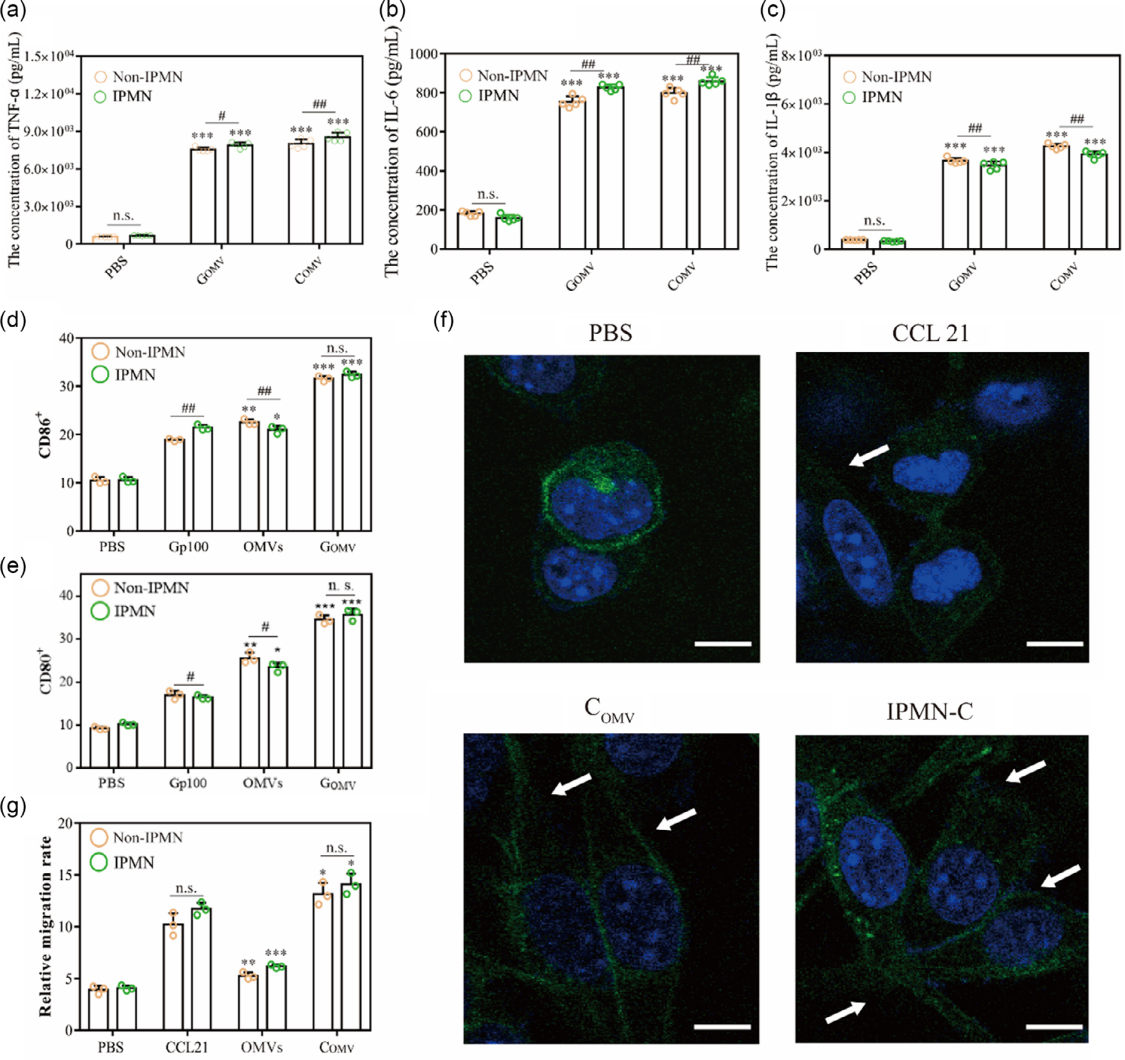
Immunogenicity of IPMN‐C in vitro. a–c) TNF‐α (a), IL‐6 (b), and IL‐1β (c) levels in cell supernatant. d,e) The expression of CD86 (d) and CD80 (e) on BMDCs. f) The distribution of C_OMV_ and IPMN‐C delivered C_OMV_ in BMDCs, Scale bar: 5 μm. g) The influence of IPMN‐C on the migration of mature DCs (*n* = 3). (*p < 0.05, **p < 0.01, ***p < 0.001, versus the PBS group. ^#^ *p* < 0.05, ^##^ *p* < 0.01, versus the IPMN group).

It is hypothesized that the applications of IPMN‐G and IPMN‐C in sequence can promote cell maturation and secrete corresponding cytokines, as well as promote the directional migration of the activated DCs to the spleen and lymph. To verify this potential regulation of IPMN‐GC in DCs, the innate immune response stimulated by IPMN‐GC was first tested by measuring the secretion of proinflammatory cytokines. It was shown that compared to the PBS group, the secretion of TNF‐α, IL‐6, and IL‐1β by BMDCs was significantly increased upon the treatment by OMVs’ involved groups (**Figure** **6**a–c).

First, the influence of IPMN on the activation of BMDCs was evaluated by measuring the expression of CD86 and CD80 on the treated BMDCs. As shown in Figure 6d,e, compared with the G_OMV_ group, the expression of CD86 and CD80 in BMDCs treated by IPMN‐G group was not significantly increased. Not only that, IPMN did not change the gp100 protein expression. The above results indicate that IPMN has good biocompatibility with a small selected voltage and has no influence on the maturation of BMDCs. In contrast, upon the treatment by IPMN‐C, the cytoskeleton changed significantly and became irregular, indicating that the cell motility potential was enhanced (Figure 6f). Real‐time cellular analysis (RTCA) experiments further investigated the effect of C_OMV_ on the migration ability of mature BMDCs. Compared with PBS group, the migration of BMDCs treated with blank OMV group did not change, while the migration ability of BMDCs in CCL21 protein group and IPMN‐C group was significantly increased by around twofold (Figure 6g).

### 2.5 In vivo Immunotherapeutic Effects of IPMN‐GC

**Figure 7:**
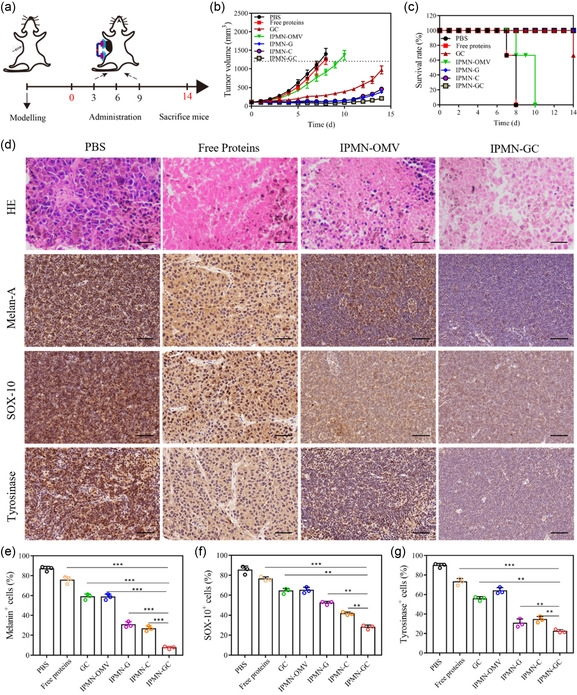
In vivo antitumor performance of IPMN‐GC. a) Schematic illustration of in vivo antitumor investigation of IPMN‐GC. b) Tumor volumes in different groups were monitored every day. c) Survival rates of mice after treatment in different groups. d) H&E staining (scale bar: 100 μm.) and histochemical analysis (scale bar: 50 μm.) of tumor in different groups. e–g) Immunohistochemistry quantitative analysis of Melan‐A, SOX‐10 and Tyrosinase in TDLN. (*p < 0.05, **p < 0.01, ***p < 0.001).

Gp100 has strong immunogenicity capability in mice and can induce definite antitumor effects. The interaction of CCL21 with CCR7 induces changes in the cytoskeleton that regulates the migration velocity of DCs. Therefore, the in vivo immunotherapeutic effect of IPMN‐GC was investigated (**Figure** **7**a). As shown in Figure 7b, compared with the free gp100 and CCL21 (protein) group, IPMN‐GC showed the strongest antitumor effect and significantly inhibited the tumor growth. The survival rates of the mice in IPMN‐G, IPMN‐C, and IPMN‐GC groups were shown on day 14 (Figure 7c). The survival rate in IPMN‐OMV reached 66%, which further demonstrated the advantage of OMVs as an immune vaccine carrier. IPMN‐G, IPMN‐C, and IPMN‐GC groups showed complete survival on day 14. Histologically, the tumor in the PBS group is dense with the tumor cells arranged dense and orderly without obvious necrosis observed. In contrast, the degree of cell necrosis in the IPMN‐GC group is obvious with the highest degree, providing further evidence for the antitumor effect of IPMN‐GC (Figure 7d).

Melan‐A, SOX‐10, and tyrosinase expression levels in the sentinel lymph node, or tumor‐draining lymph nodes (TDLN), are the major indicators of metastasis of melanoma. As shown in Figure 7e and S6, Supporting Information, compared with the free protein group, the contents of Melan‐A in lymph nodes in GC, IPMN‐OMV, IPMN‐G, IPMN‐C, and IPMN‐GC groups were decreased significantly, of which Melan‐A in IPMN‐GC group reached the lowest level of 8%. The results of SOX‐10 and tyrosinase are similar to that of Melan‐A (Figure 7f,g). These results give evidence that the combined use of MN and iontophoresis could significantly enhance the antitumor and metastasis prevention effects. Furthermore, with the sequential delivery of G_OMV_ and C_OMV_ by IPMN, these antitumor effects can be maximized to the highest degree.

**Figure 8:**
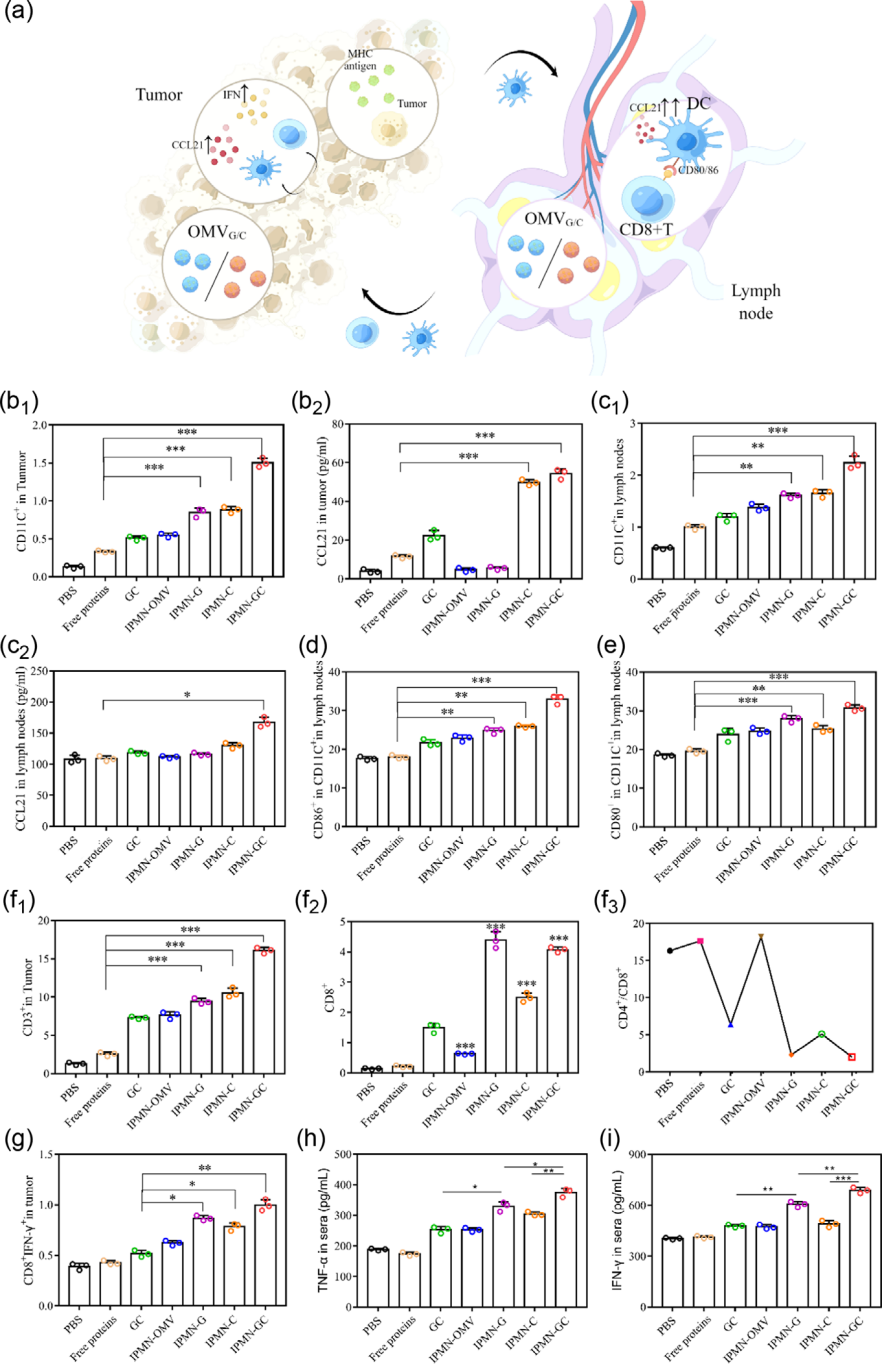
In vivo antitumor mechanism of IPMN‐GC. a) Schematic of the antitumor mechanism of IPMN‐GC through immunotherapeutic effects. b) The number of DCs (b_1_) and CCL21 concentration (b_2_) in tumor. c) The number of DCs (c_1_) and CCL21 concentration (c_1_) in lymph node. d,e) The expression of CD86 (d) and CD80 (e) in lymph nodes. f) Percentage and classification of T lymphocytes at tumor sites. (f_1_: CD3, f_2_: CD8, f_3_: CD4/CD8). g) The percentage of CD8^+^ IFN‐γ^+^ cells in tumor. h,i) Serum concentration of TNF‐α and IFN‐γ. (*p < 0.05, **p < 0.01, ***p < 0.001, *n* = 3).

The antitumor performance exhibited by IPMN‐GC treatment reminded us to investigate the underlying molecular mechanisms (**Figure** **8**a). As shown in the Figure 8b,c and S7, S8, Supporting Information compared with the free protein group, the CCL21 concentration was shown to be increased significantly in the IPMN‐GC group, leading the number of DC cells to be increased by 4.1 times in the tumor site. A similar influence was identified in lymph nodes, indicating that the IPMN system can promote the enrichment of OMVs in tumors and lymph nodes, which promoted the migration of DC cells with the immunotherapeutic effects enhanced. The influence of IPMN‐GC on the maturation of DCs in lymph nodes was then investigated. As shown in Figure 8d,e and S9, Supporting Information, compared with IPMN‐G and IPMN‐C, IPMN‐GC groups significantly increased the percentages of CD86^+^ and CD80^+^ T cells, indicating that the sequential delivery of IPMN‐G and IPMN‐C can significantly promote DC maturation in TDLN. Lymphocytes are important cellular components of the immune response function of the body and are the main executors of the immune function of lymphatic system. As shown in Figure 8f_1_ and S10, S11, Supporting Information, compared with the free protein group, IPMN‐GC significantly increased the numbers of CD3^+^ T cells in tumors and TDLN. To evaluate the effect of IPMN‐GC on cellular immunity, the CD4^+^/CD8^+^ ratio was investigated. As shown in Figure 8f_2_ and S12, Supporting Information, IPMN‐GC groups can increase the percentages of CD8^+^ and CD4^+^ T cells. As shown in Figure 8f_3_, compared with free protein, IPMN‐GC groups significantly reduced the CD4^+^/CD8^+^ ratio, indicating that IPMN can effectively promote the effect of cellular immunity of G_OMV_ and C_OMV_ in vivo and has the strongest ability to enhance MHC class I antigen presentation and cellular immune response. Activated lymphocytes are the main effector cells for antitumor performance, and interferon gamma (IFN‐γ) supports effector responses of CD8^+^ cytotoxic T lymphocytes (CTLs). From Figure 8g, among them, the content of activated CTLs in IPMN‐GC group was the highest, indicating that IPMN‐GC could significantly enhance the immune response of T cells for cancer inhibition. As shown in Figure 8h,i, compared with IPMN‐G and IPMN‐C, IPMN‐GC can significantly promote IFN‐γ. The above results indicate that IPMN‐GC can stimulate the production of a strong immunological regulation for antitumor performance.

### 2.6 In Vivo Immunoprophylaxis Efficacy of IPMN‐GC

**Figure 9:**
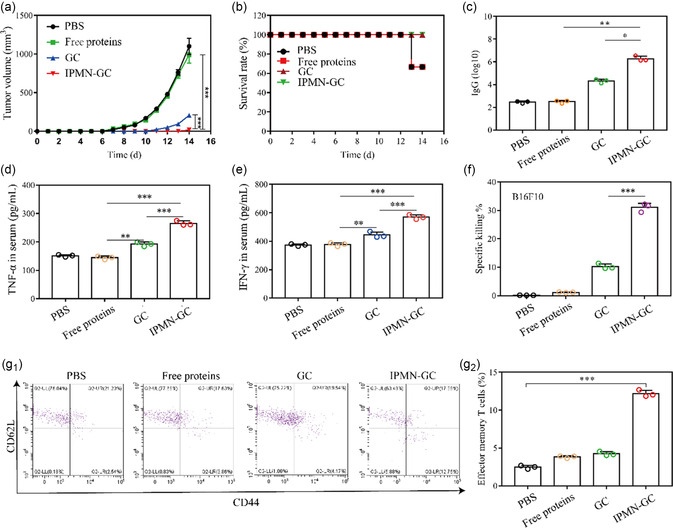
In vivo immunoprophylaxis efficacy of IPMN‐GC. a) Tumor volumes in different groups were monitored every day (***p < 0.001). b) Survival rates of the tested mice in different groups. c) The titer of IgG in serum. d) The concentration of TNF‐α in serum. e) Serum concentration of IFN‐γ. f) The specific killing ability of splenocytes to B16F10. g_1_) The percentage of effector memory T cells (CD3^+^CD8^+^CD62L^‐^CD44^+^) by fluorescence‐activated cell sorting (FACs). g_2_) Quantitative analysis of effector memory T cells. (*p < 0.05, **p < 0.01, ***p < 0.001, *n* = 3).

Using B16 tumor‐bearing C57BL/6 mice as animal models and PBS as the control group, the immunoprophylactic effect of IPMN‐GC on melanoma was evaluated. As shown in **Figure** **9**a, tumors in PBS and free protein group showed a rapid growth trend, reaching a volume of about 1100 mm^3^ on day 14. In contrast, tumor growth was inhibited in GC group, indicating that OMVs had a transdermal property and partially exerted the immunoprophylaxis effect. IPMN‐GC significantly inhibited tumor growth with only 2 mice developed tumors on day 14. The survival rate of mice is shown in Figure 9b. Mice in the GC group and IPMN‐GC group survived on day 14. The above results indicate that IPMN‐GC has a high immunoprophylactic effect in vivo. The concentration of specific antibodies in vivo is the main index for evaluating humoral immunity and immunoprophylaxis. As shown in Figure 9c, compared with the free protein group, IPMN‐GC treatment produced significantly higher titers of IgG antibodies. Furthermore, compared with free protein, IPMN‐GC can significantly promote the secretion of IFN‐ γ and TNF‐ α, which are important antitumor biofactors (Figure 9d,e). These results give evidence of the efficacy of IPMN‐GC treatment in stimulating the body to produce strong and long‐term antitumor immunity. Gp100‐pulsed BMDCs may induce specific cytotoxicity of T lymphocytes against B16F10 cells. The specific cytotoxicity of splenic lymphocytes was therefore tested. As shown in Figure 9f, compared with the GC (transdermal application of G_OMV_ and C_OMV_ with a 12 h interval) group, IPMN‐GC group significantly increased the killing effect on B16F10 cells with the highest efficacy around 30%. And the killing effect of IPMN‐GC on MCF‐7 cells had no significant difference (Figure S13, Supporting Information). These results indicate that IPMN‐GC can effectively induce antigen‐specific CTLs. When stimulated by antigen, effector memory T cells can quickly produce effector cytokines for immune protection. As shown in Figure 9g_1_, g_2_, compared with the free protein and GC groups, the IPMN‐GC group significantly increased the content of effective memory T cells in the spleen, indicating that IPMN‐GC is a potential immunoprophylactic delivery system.

### 2.7 Biosafety of IPMN‐GC

Along with the increased body weight of the tested animals, neither sign of toxicity nor side effect was observed in the tested animals (Figure S14, Supporting Information), the H&E stain revealed that the in vivo application of all tested regimens did not make influence to the organs (Figure S15, Supporting Information). All of these results demonstrated that IPMN‐GC is safe and well‐tolerated for in vivo bioapplications, indicating the long‐term good safety of IPMN‐GC in vivo.

## 3 Discussion

Immune activation using vaccines is one of the most important strategies for cancer immunotherapy.^[^ ^22^ ^]^ However, up to now, vaccines are mainly administered by intramuscular (i.m.) or subcutaneous (s.c.) injection, which has apparent limitations such as pain and stress, needle‐related injuries, and contradictious, the requirement of trained personnel to be overcome.^[^ ^23^ ^]^ Given its high accessibility and unique immune‐eliciting capacities, TCI referring to vaccination through the transdermal delivery of vaccination therapeutics instead of syringe‐based injection has drawn attention for centuries since the first record of vaccination against smallpox in 1796.^[^ ^24^ ^]^ However, the TCI requires not only the vaccine protein as a core driver but also the drainage and presentation of chemotactic protein to simulate the migration of mature DCs to lymph node.^[^ ^25^ ^]^ The combinational delivery of both antigen and chemokine proteins is therefore very critical to induce the vigorous TCI immune response; however, it is kept to be verified for the efficacy of this concept‐to‐proof transcutaneous immunotherapy with an efficient transdermal delivery platform.

Besides the efficient transdermal vehicle, to enhance the cellular uptake and bioavailability of biomacromolecules in skin DCs, nanovectors such as liposomes,^[^ 8, 26 ^]^ polymer micelles,^[^ 27, 28, 29 ^]^ and inorganic nanoparticles^[^ ^30^ ^]^ have been widely investigated as vehicles. However, the problems along with synthetic nanoparticles, such as the unwanted immune response, the potential toxicity of synthetic materials to cells and tissues, as well as the difficulty in reproducing the high entrapment efficiency for external protein, have greatly limited the therapeutic effects of the nanovaccine‐based transcutaneous immunotherapy.^[^ ^31^ ^]^ In our previous study, we demonstrated transgenic micro‐organisms as novel molecular farms to produce OMVs with target proteins incorporated, which are the natural and efficient nanovehicles to entrap and deliver protein drugs with enhanced cellular uptake and tissue penetration efficacy.^[^ ^13^ ^]^ In this study, OMVs derived from antigen or chemokine genes transformed *E. coli* and were entrapped into iontophoresis‐driven MN to form triplatforms (named IPMN‐G and IPMN‐C, respectively). Superior infiltration and uptake by skin DCs of G_OMV_ and C_OMV_ followed by enhanced migration of mature DCs to lymph nodes were obtained, showing excellent transdermal delivery efficiency.

IPMN‐G and IPMN‐C not only circumvent the susceptibilities and random transport of proteins in conventional delivery strategies by directly delivering the proteins into the skin melanoma site through noninvasive MN but also obviously enhance the uptake and migration of activated skin DCs toward the immunological organs by driving the protein‐secreting OMVs by iontophoresis with biomimetic intensity. Cancer immunotherapy induced by the transdermal applications of IPMN‐G and IPMN‐C in sequence showed an obvious retardation of the progression, relapse, and prevention of skin melanoma and metastasis.

## 4 Conclusion

IPMN‐GC‐based triplatform provides a proof‐of‐concept strategy in engineering bacterial‐derived OMV‐loaded MN patch driven by iontophoresis into a transcutaneous vaccine for the transdermal delivery of antigen and chemokine in sequence with great application potential in cancer immunotherapy, as well as many other dermatological diseases.

## 5 Experimental Section

5.1

5.1.1

##### Cell Lines and Animals

B16F10 cells were obtained from Shanghai Institute for Biological Sciences, CAS (Shanghai, China) and cultured in DMEM with 10% v/v fetal bovine serum at 37 °C in a humidified 5% CO_2_ incubator. BMDCs were derived from bone marrow of the femur and tibia of C57BL/6 mice following published protocols.^[^ ^32^ ^]^ In brief, cells collected from bone marrow were suspended in RPMI supplemented with 10% FBS, 2 mm glutamine, 1 μg mL^−1^ streptomycin, 1‐unit mL^−1^ penicillin, 20 ng mL^−1^ mouse recombinant GM‐CSF, and 10 ng mL^−1^ mouse recombinant IL‐4, following RBC lysis and washing and cultured in plates under normal tissue culture ambiance. On the 3rd day, 50% of the culture medium was replaced by fresh medium and on the 7th day, cells were harvested for experiments. Cells were harvested and incubated with normal murine serum for 30 min on ice to block Fc receptors and inhibited nonspecific staining. After 2 rounds of PBS washing, 1 × 10^6^ cells/sample were incubated for 1 h on ice with combinations of the FITC‐anti‐mouse CD11c antibodies (1:30 dilution). Surface fluorescence was measured on a Cytomic FC 500MCL (Beckman Coulter) flow cytometer. C57BL/6 (four weeks old) were purchased from Shanghai SLAC Laboratory Animal Co. Ltd., China. All animals were maintained under constant conditions(temperature 25 ± 1 °C) and had free access to a standard diet and drinking water. All animal experimental procedures were performed in obedience to the guidelines and protocols of the Animal Experimental Ethics Committee of Zhejiang University.

##### Reagents and Antibodies

BCA protein assay kit, methylthiazoletetrazolium (MTT), and actin‐tracker green‐488 were purchased from Beyotime Biotechnology Inc. (Nantong, China). Chlorpromazine (CPZ), methylated‐β‐cyclodextrin, indomethacin, and 40,6‐diamidino‐2‐phenylindole (DAPI) were purchased from Sigma‐Aldrich (Steinheim, Germany). FITC‐anti‐mouse CD3, APC‐anti‐mouse CD8α, PE/Cy7‐anti‐mouse IFN‐γ, FITC‐anti‐mouse CD11c, PE/Cy7‐anti‐mouse CD80, APC‐anti‐mouse CD86 were purchased from Invitrogen (USA). FITC‐DNA, cy5.5‐DNA, and cy5.5‐EHD2 monoclonal antibodies were purchased from Invitrogen (Carlsbad, CA, USA). The tumor necrosis factor α (TNF‐α) ELISA kit, interleukin‐6 (IL‐6) ELISA kit, and interleukin‐1beta (IL‐1β) ELISA kit were purchased from eBioscience (USA). Poly‐glycidyl methacrylate (PGMA), trimethylolpropane trimethacrylate, triethylene glycol dimethacrylate, 2‐methoxyethanol, and Irgacure 184 were purchased from Aladdin Biochemical Technology Co., Ltd (Shanghai).

##### Preparation and Characterization of OMV from Transgenic E. coli

Plasmid DNAs (pDNAs) encoding human gp100 (gp100) and mouse CC chemokine ligand 21 (CCL21) (Sangon Biotechnology Inc., China), respectively, were transformed into *E. coli* by heat shock method, forming gp100 transformed *E. coli* (G‐*E. coli*) and CCL21 transformed *E. coli* (C‐*E. coli*). Briefly, 100 μL of competent *E. coli* were mixed with 1 μL of pDNAs (gp100 or CCL21) (0.1 μg μL^−1^) and incubated in ice bath for 30 min. After that, the mixture was heated at 42 °C for 100 s. Following the heat‐shock step, the mixture was kept on ice for another 6 min. Subsequently, these bacteria were supplied with fresh LB‐Broth‐Medium and incubated in a shaker at 37 °C for 1.5 h. Next, the resulting culture was spread on an agar‐LB plate containing kanamycin (50 μg mL^−1^) or ampicillin (100 μg mL^−1^) and incubated with shaking for 18 h at 37 °C. The survived G‐*E. coli* and C‐*E. coli* clones were harvested and further cultured in LB medium containing kanamycin or ampicillin to an optical density of 0.6 at 600 nm. Then, an appropriate amount of culture was transferred to a fresh LB medium containing kanamycin or ampicillin and incubated overnight at 37 °C with shaking. After the expanded cultivation of G‐*E. coli* and C‐*E. coli,* G_OMV_ and C_OMV_ were collected from the bacterial culture supernatant by ultracentrifuge (OptimaTM MAX‐XP, USA). The collected OMVs were then filtered through a 0.22 μm filter (Thermo Scientific) and purified using 100 KDa cutoff ultrafiltration membranes (Millipore). The particle size and zeta potential of OMVs were measured by Zetasizer Nano ZS90 (Malvern instruments, UK). The morphology of OMVs was observed by a transmission electron microscope (TEM, JEM‐2100). DiI‐OMVs were tracked using CLSM (Olympus, Japan). Gp‐100 peptide in the G_OMV_ and CCL21 protein in the C_OMV_ was assessed by western blot (WB) analysis, high‐performance liquid chromatography (HPLC), and ELISA (BOSTER, China).

##### Western Blot Analysis

To verify the embedding of gp100 protein and CCL21 in OMVs, both the prepared OMVs, G‐*E. coli* and C‐*E. coli* strains were identified using WB analysis. Briefly, the total bacterial protein was extracted using the total protein extraction kit (Protease Inhibitor Cocktail). BCA Protein Assay Kit (ab102536) was used for total protein quantification. The primary antibodies used were anti‐melanoma gp100 (Abcam) and anti‐CCL21 (Abcam). The protein bands were detected with secondary antibodies conjugated to horseradish peroxidase (HRP) and enhanced chemiluminescence. Films were scanned by SuperSignal West Dura Extended Duration Substrate. Glyceraldehyde‐3‐phosphate dehydrogenase expression was used as a loading control.

##### Fabrication and Characterization of OMV‐Loaded Microneedles Array

MN made of poly‐glycidyl methacrylate were prepared by the combination of molding and porogen methods. Briefly, a female mold for MN was made with polydimethylsiloxane (PDMS) with two stock solutions by following a previously reported protocol. The monomer stock solution was prepared by mixing the monomer glycidyl methacrylate (10 mL), crosslinker trimethylolpropane trimethacrylate (5 mL), and crosslinker triethylene glycol dimethacrylate (15 mL). A porogen stock solution was prepared by dissolving poly(ethylene glycol) (10 kDa) in 2‐ methoxyethanol at 65 °C at the weight ratio of 1:5. Just after mixing the monomer and porogen stock solutions (1:1 in volume), the photoinitiator Irgacure 184 was added (1 wt% to the monomer), followed by pouring the mixture into the PDMS mold. The mold with the mixture was put under vacuum at ≈1.0 × 10^2^ Pa for 40 min to ensure that bubbles were removed and all the cavities of the mold were filled with the solution. Photopolymerization was conducted by irradiation of 365 nm UV light for 1 h to make MN array chip composed of 144 needles with an interval of 0.2 mm on a planar substrate (ϕ10 × 0.8 mm in thickness). By dissolving the porogen with 60 °C ethanol/water (1:1 volume) for 24 h, the naked MNs were obtained. The porosity of the naked MN was estimated as ≈*P* = (1 − *m*_2_/*m*_1_) × 100%, and the mass of the solid MN and porous MN was recorded as *m*_1_ and *m*_2_, respectively. Then, 50 μL of G_OMV_ and C_OMV_ (2 mg mL^−1^) were dropped on MNs, respectively, and centrifuged for 10 min at 500 rpm to obtain MN‐G_OMV_ and MN‐C_OMV_. The micromorphology of MNs was observed by scanning electron microscopy (SEM). The section of MN‐OMV was scanned by SEM X‐ray energy‐dispersive spectroscopy (SEM–EDS) to study the element distribution and analyze the distribution of OMVs on MNs.

##### Construction of the Iontophoresis‐Driven MN Array

Take MN‐G_OMV_ as an example, the MN‐G_OMV_ and blank MN were respectively coated with graphite electrodes. The negative pole of the direct current power supply was connected to the MN‐G_OMV_, and the positive pole was connected to the blank MN for constructing IPMN‐G. The construction and application of IPMN‐C were consistent with those of IPMN‐G. 3D printing technology was used to prepare a scaffold mold to support MNs for in vitro experiments. First, we used the proe software to conduct 3D design and modeling. Then, fused deposition modeling was used to conduct 3D printing. The filamentous thermoplastic material was heated and melted by the nozzle. Under the control of the computer, the nozzle and worktable moved in the *X*‐ and *Y*‐axis directions, respectively, and the material in the molten state was extruded and finally solidified through the accumulation of materials layer by layer to form the product. (The 3D scaffold is shown in Figure 3g).

##### Drug Release Test

Take IPMN‐G as an example, MN‐G_OMV_ and blank MN were on two 3D scaffolds, respectively. Then 6 V direct‐current voltage was applied by a direct‐current power source for 30 min. After that, the power supply was removed and the amount of released gp100 protein at 0.5 h was determined by ELISA. Then, the drug‐loaded MN was pipetted into a dialysis membrane and submerged in PBS. Dialysates were collected at predetermined time points. The amount of released gp100 protein was determined by ELISA. The test procedures of IPMN‐C were consistent with those of IPMN‐G.

##### Skin Penetration Assay

To test in vitro skin penetration of IPMN‐GC, C57BL/6 mice (four weeks old) were anesthetized and the dorsal skins were depilated. The skins were applied with free DiI‐BSA, DiI‐OMV, and IPMN‐OMV (20 μg OMV per mouse), respectively. In the IPMN‐OMV group, the IPMN‐OMV was removed after being applied onto the skin for 30 min at the 6 V direct current voltage. After 6 h, the mice were sacrificed by cervical dislocation and the dorsal skin administrated was collected. Then, the skin was washed and prepared into the paraffin and sliced into 6 μm thick by standardized protocols. The depth of the penetrated OMVs was observed by fluorescence microscopy. To testify the transdermal efficacy of IPMN‐GC, the skins of C57BL/6 mice were used with Franz diffusion cells. Franz diffusion cells with an effective diffusion area of 0.6 cm^2^ and receptor volume of 5.0 mL were used. The total thickness of the skin was washed three times with 1.0 mL of water and mounted between the two halves of the cell with the SC facing the donor compartment. The receptor compartment was filled with 5.0 mL of PBS and checked to ensure there were no air bubbles between the receptor fluid (PBS) and the skin. The experiments were repeated in triplicate for a total of 24 exposed cells and two blanks. The FDC was maintained at a constant temperature of 32 ± 1 °C through thermostatic bath circulation. At fixed time intervals (0.5, 1, 2, 4, 8, 10, and 24 h), aliquots were collected from the receptor chambers and immediately replaced with the same volume of fresh PBS medium. The magnetically stirring speed was 300 rpm. The OMV solutions and IPMN‐OMV (20 μg OMV per mouse) were added to each donor cell. Samples (100 μL) were withdrawn from the diffusion chamber at different intervals (0.5, 1, 2, 4, 8, 10, and 24 h), and fresh PBS was replenished. The amount of gp100 and CCL21 protein in the diffusion chamber was determined by ELISA kit.

##### In Vitro Biocompatibility of IPMN Arrays

The in vitro cytotoxicity of IPMN‐G and IPMN‐C to skin cells was assayed. Briefly, BMDCs (3 × 10^5^ cells/well) were seeded on a six‐well plate and cultivated for 12 h. Then PBS (control) or IP with different voltage‐driven MN‐G or MN‐C were added to cells for 30 min at 37 °C. The cells were incubated for another 18 h and washed with PBS twice. The culture medium was replaced with fresh one containing 0.5 mg mL^−1^ 3‐[4,5‐dimethyl‐thiazolyl‐2]‐2,5‐diphenol tetrazolium bromide (MTT). After 4 h, the supernatant was aspirated, and 200 mL of dimethyl sulfoxide (DMSO, Sigma, USA) was added to each well. The plate was micro‐oscillated for 30 s, and then the absorbance at 570 nm was measured with a microplate reader. The cell viability was normalized to that of nontreated cells. Five samples were examined from each group, and the cell viability was determined.

##### Endocytosis and Intracellular Pathway of IPMN‐Delivered OMVs in Cells

BMDCs were seeded on six‐well culture dishes at a cell density of 3–5 × 10^5^ cells per well. Half medium was replaced every 2 d. On day 6, nonadherent cells were collected for further investigation. To directly observe the cellular uptake of G_OMV_ at different time points, Dil‐G_OMV_ were incorporated into the MNs, forming the MN‐Dil‐ G_OMV_, which were tracked by CLSM and flow cytometry to indicate the endocytosis and intracellular pathway of Dil‐G_OMV_. After cells were incubated overnight, the G_OMV_ were added to each dish under the IPMN and observed at 12 and 24 h, respectively. For CLSM observation, cellular lysosomes were labeled with Lyso‐Tracker Red (Beyotime) and nuclei were stained with 4,6‐diamidino‐2‐phenylindole (DAPI, Keygen). Intracellular location of G_OMV_ at different time points was observed under laser scanning confocal microscopy (IX81‐ FV1000, Olympus). For flow cytometry, cells were detached with 0.25% trypsin–EDTA. FACS running buffer (500 μL), consisting 98% PBS and 2% FBS, was added to each well. Cells were then transferred to a FACS tube with a filter lid, and the Dil (from OMVs) signals were acquired on a Cytomic FC 500MCL (Beckman Coulter) flow cytometer. At least 10 thousand cells were analyzed for each sample. To elucidate the mechanism of cellular uptake, BMDCs were seeded in the 12‐well plates with a density of 1 × 10^6^/well in serum‐free medium overnight. Then cells were either untreated or pretreated with 4 °C (energy‐mediated endocytic inhibitors), chlorpromazine (CPZ) (10 μg mL^−1^ clathrin‐mediated endocytic inhibitors), methyl‐β‐cyclodextrin (50 μg mL^−1^ phagocytosis‐mediated endocytic inhibitors), and indomethacin for 30 min, respectively. Cells were incubated with IPMN‐G for 6 h. Then, the medium was replaced by RPMI‐1640 with 10% FBS. After another culture of 18 h, mean fluorescence intensity per cell was measured by examining using flow cytometry (BD Accuri C6, BD Biosciences, USA).

##### Binding of IPMN Released OMVs to BMDCs

Membranes of the mature BMDCs and C_OMV_ were labeled with CM‐Dil and DiO, respectively. The mature BMDCs processed by C_OMV_ were inoculated on a 24‐well plate at the cell density of 1 × 10^5^ cells/well and cultured overnight at 37 °C and 5% CO_2_, respectively. The mature BMDCs were incubated with C_OMV_ (20 μg mL^−1^) or IPMN‐C (20 μg C_OMV_) for 1, 3, and 6 h. 4% paraformaldehyde solution (w/w) was fixed at room temperature for 15 min. The fixed solution was discarded, and the PBS was flushed three times. CLSM was used to observe the distribution and possible binding mechanism of DiO‐labeled C_OMV_ and BMDC.

##### Influence of IPMN‐OMVs Treatment in the Behavior of BMDCs

Cell migration includes a series of behavior including the extension of cell front protrusion (including plate‐like pseudopodia and filamentous pseudopodia), formation of new cell front adhesion, and the movement of cell body. To identify the influence of IPMN‐C in the migration of BMDCs, we observed these processes by the CLSM. Particularly, mature BMDCs were inoculated at the cell density 1 × 10^5^ cells/well on a six‐well plate and cultured overnight at 37 °C and 5% CO_2_. The mature BMDCs were incubated with PBS, CCL21 protein (10 ng mL^−1^), C_OMV_ (20 μg mL^−1^), or IPMN‐C (containing 20 μg C_OMV_). After a further 6 h‐incubation, cells were then harvested and fixed with 4% paraformaldehyde for 15 min at room temperature. The cells were then stained with 5 μL of FITC‐labeled ghost pen cyclic peptide solution for 30 min in the dark at room temperature. Finally, the changes of BMDCs skeleton were analyzed by CLSM to indicate the influence of IPMN‐C on BMDCs migration.

RTCA was used to evaluate the influence of IPMN‐C in BMDCs migration. The cell density of 10^5^ cells/well was inoculated on a 24‐well plate, cultured overnight at 37 °C and 5% CO_2_, and then replaced with a serum‐free medium for culture. The mature BMDCs were digested by trypsin and collected. Zero adjustment control group: 165 μL was added to the lower chamber serum‐free medium. Experimental group: 145 μL was added to the lower chamber serum‐free medium and 20 μL 10 ng mL^−1^ CCL21 protein. 30 μL was added to the upper room of each group serum‐free medium, stood for 1–2 h at 37 °C and 5% CO_2_, and the baseline was measured. Then 100 μL BMDCs suspension was added treated with PBS, CCL21, and OMV and C_OMV_ was placed on RTCA Station after being placed in the ultraclean platform at room temperature for 0.5 h. It was automatically measured every 5 min at 37 °C and 5% CO_2_ for 24 h.

##### In vivo Antitumor Performance of IPMN‐GC

B16F10 cells (1 × 10^6^ cells per mouse) were inoculated subcutaneously at right flank of male C57BL/6 mice (4–5 weeks, 20 g per mouse, *n* = 6) mice to set up the melanoma model. Mice were randomly divided into seven groups and topically administrated with PBS, free proteins (0.2 μg gp100 kg^−1^ and 0.2 μg CCL21 /kg mouse), GC (0.5 mg G_OMV_ /kg and 0.5 mg C_OMV_ /kg mouse), IPMN‐OMVs (1 mg OMV/kg mouse), IPMN‐G (1 mg G_OMV_ /kg mouse), IPMN‐C (1 mg C_OMV_ /kg mouse), and IPMN‐GC (0.5 mg G_OMV_ /kg and 0.5 mg C_OMV_ /kg mouse), respectively. Animals used for evaluating the antitumor effects of OMVs were immunized on days 3, 6, and 11 after the primary tumor volumes reached a size of 50 mm^3^. The MN loaded with vesicles was applied at the tumor site, and the blank MN patch was applied right next to the periphery. Mice were sacrificed and tumors were collected on day 17. Tumors were then digested into a single‐cell suspension for evaluating the infiltration of immune cells (*n* = 4). Peripheral blood and splenocytes were collected on day 14 to analyze antigen‐specific CD8^+^ T cells by flow cytometry analysis. The survival experiment was terminated when tumor volume reached 1500 mm^3^ (*n* = 6). The tumor volumes and body weights of mice were recorded from the first day until the end of the experiment. The tumor volume was calculated using the following formula: width^2^ × length × 0.5. The survival rates of mice were recorded until day 17 post‐treatment. All the major organs of the tested animals were collected and examined by H&E staining.

##### In vivo Immunoprophylaxis Efficacy of IPMN‐GC

Male C57BL/6 mice (4–5 weeks, 20 ×g per mouse, *n* = 6) were randomly divided into seven groups and topically administrated with PBS, free proteins (0.1 mg gp100 kg^−1^ and 0.1 mg CCL21 /kg mouse, 50% of the concentrations of gp100 within G_OMV_ and CCL21 within C_OMV_), GC (0.5 mg G_OMV_ /kg and 0.5 mg C_OMV_ /kg mouse), IPMN‐OMVs (1 mg OMV/kg mouse), IPMN‐G (1 mg G_OMV_ /kg mouse), and IPMN‐C (1 mg C_OMV_ /kg mouse), and IPMN‐GC (0.5 mg G_OMV_ /kg and 0.5 mg C_OMV_ /kg mouse), respectively. Animals used for evaluating immunoprophylaxis of OMVs were plant B16F10 cells (1 × 10^6^ cells per mouse) 30 d after IPMN‐GC administration. The placement of the MN was consistent with the antitumor experiment in vivo. Mice were sacrificed and tumors were collected on day 14. Tumors were then digested into a single‐cell suspension for evaluating the infiltration of immune cells (*n* = 6). Peripheral blood and splenocytes were collected on day 14. Splenocytes were further used for the specific killing assays. Splenocytes were cultured with antigen (gp100) overnight and then cultured with B16 cells and MCF‐7 cells at the ratio of 10:1 for 24 h. Nonadherent cells were removed, and adherent cells were washed with PBS. CCK‐8 assay was used to calculate the percent of specific killing. The tumor volumes and body weights of mice were recorded from the first day until the end of the experiment. The tumor volume was calculated using the formula: width^2^ × length × 0.5. The survival rates of mice were recorded until day 17 post‐treatment. All the major organs of the tested animals were collected and examined by H&E staining.

##### Histology and Immunohistochemistry Analysis

Histological analysis was performed by H&E staining. For immunohistochemistry, tumor slices were embedded in paraffin. Immunohistochemistry was performed by deparaffinization, antigen retrieval, permeabilization, and blocking in 5% BSA. The primary antibodies used were anti‐SOX‐10, anti‐Tyrosinase, and anti‐Melanie‐A (Abcam). Secondary antibodies conjugated to HRP were used. After counterstaining with hematoxylin staining, the images were taken using a microscope (Eclipse Ti‐S, Nikon).

##### Statistical Analysis

Data are expressed as mean ± standard deviation. For comparisons between two groups, means were compared using unpaired two‐tailed student's *t*‐tests. The one‐way analysis of variance with post‐hoc Tukey's honest significant difference was conducted for multiple sample analyses. All statistical analyses were performed using GraphPad Prism version 8 software (GraphPad Software Inc).

## Conflict of Interest

The authors declare no conflict of interest.

## Author Contributions

L.P. took care of conceptualization and conceived the project, project administration, and supervision. M.W. and W.X performed the experiments and conducted data analysis. L.P., G.Y., Q.X., N.Z., and H.C. wrote and edited the manuscript. All authors discussed the results and commented on the manuscript.

## Supporting information

Supplementary Material

## Data Availability

The data that support the findings of this study are available from the corresponding author upon reasonable request.
