# Supplementary material for: Iontophoresis‐Driven Microneedle Arrays Delivering Transgenic Outer Membrane Vesicles in Program that Stimulates Transcutaneous Vaccination for Cancer Immunotherapy
Source: Small Sci. 2023 Oct 2;3(11):2300126. doi: 10.1002/smsc.202300126 (PMC11935981; doi:10.1002/smsc.202300126)
Supplement: Supplementary file 1 — Supplementary Material [file SMSC-3-2300126-s001.pdf]

Supplementary Materials for

Iontophoresis-Driven Microneedle Arrays Delivering Transgenic Outer Membrane Vesicles in Program Stimulates Transcutaneous Vaccination for Cancer Immunotherapy

Maoze Wang[1#], Ge Yan[1#], Qiyao Xiao[1], Nan Zhou[1], Hao-Ran Chen[1], Wei Xia[4], Lihua Peng[1,2,3]*

[1]College of Pharmaceutical Sciences, Zhejiang University, Hangzhou, 310058, PR China

[2]Jinhua Institute of Zhejiang University, Jinhua 321299, Zhejiang, PR China.

[3]State Key Laboratory of Quality Research in Chinese Medicine, Macau University of Science and Technology, Macau, PR China

[4]Division of Applied Materials Science, Department of Materials Science and Engineering, Uppsala University, Uppsala, Sweden

[#]These authors contributed equally to this work.

*Address correspondence to

Li-Hua Peng, Ph.D, Associate Professor.

Email: lhpeng@zju.edu.cn

Tel/Fax: +86-571-88981231

College of Pharmaceutical Sciences, Zhejiang University, 866# Yuhangtang Road, Hangzhou, 310058, P.R. China.

This PDF file includes: Figures. S1 to S15

Figure. S1.

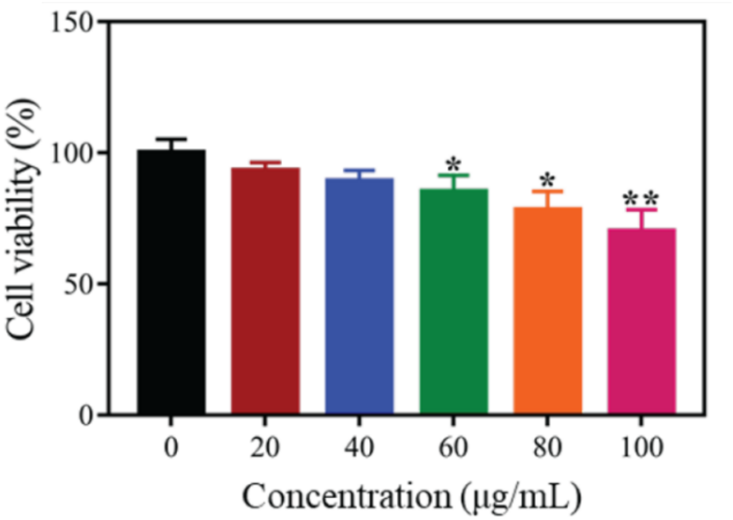

Supplementary Fig.1 Cells viability of DC upon the treatment by the detoxified OMVs with 20% antibiotics (w/w). (*p < 0.05, **p < 0.01, ***p< 0.001 n = 3).

Figure. S2.

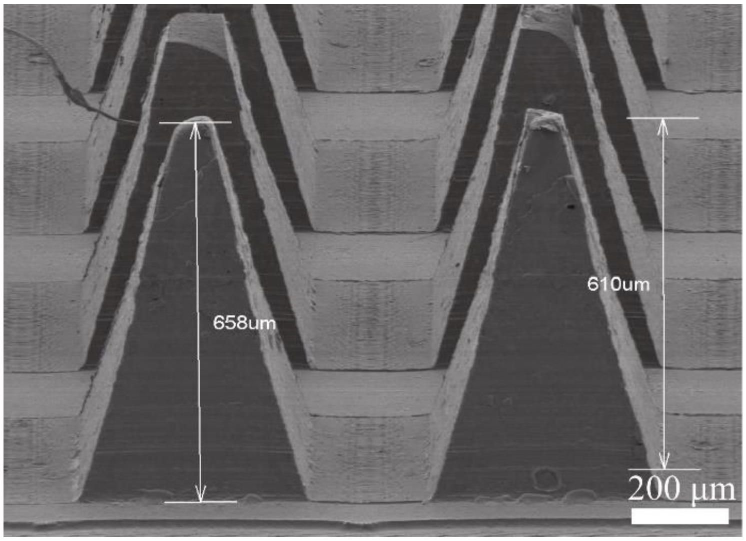

Supplementary Fig.2 SEM images of MNs.

Figure. S3.

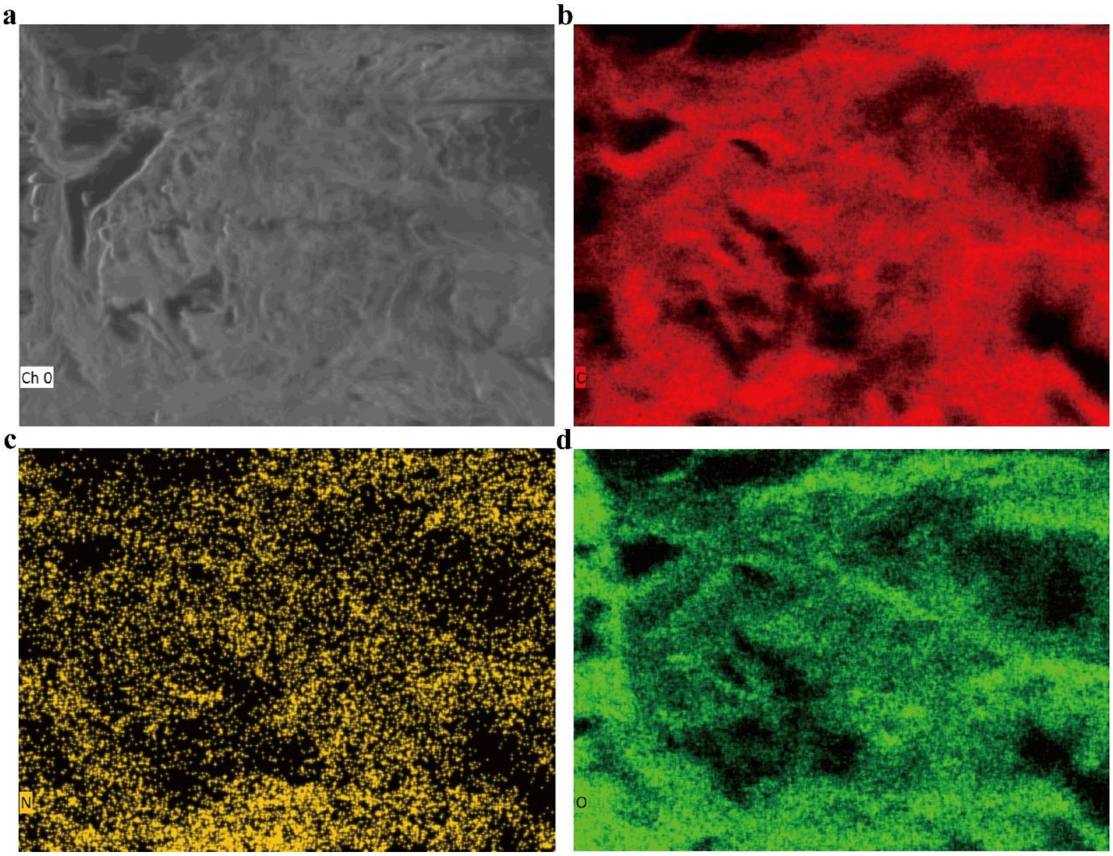

Supplementary Fig.3 EDS analysis of MN-OMVs. Red indicates carbon element, yellow indicates nitrogen element and green indicates oxygen element.

Figure. S4.

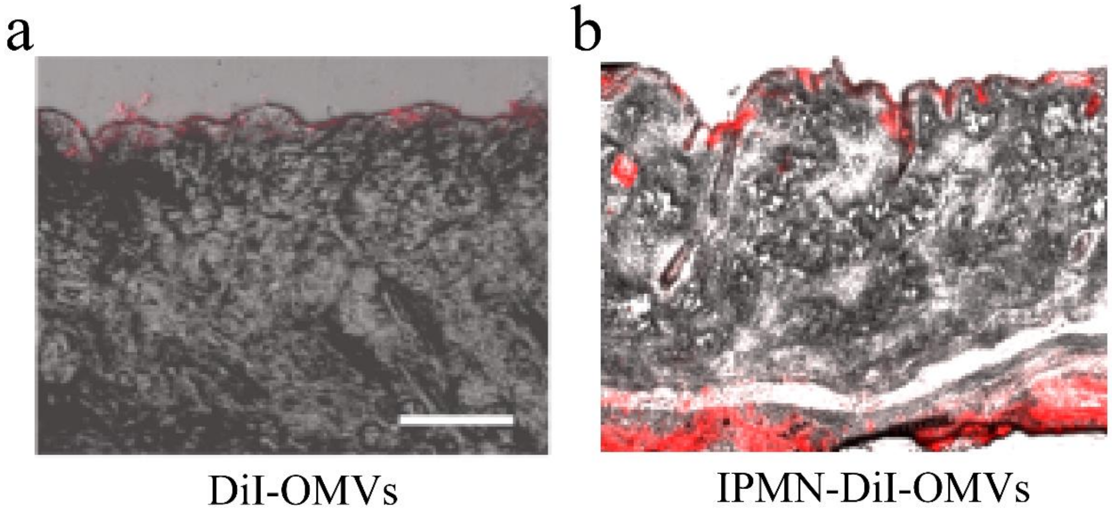

Supplementary Fig.4 Transdermal effect of IPMN-OMV. 24 h after administration, skin sections were observed with CLSM; red: Dil. Scale bar: 100 μm.

Figure. S5.

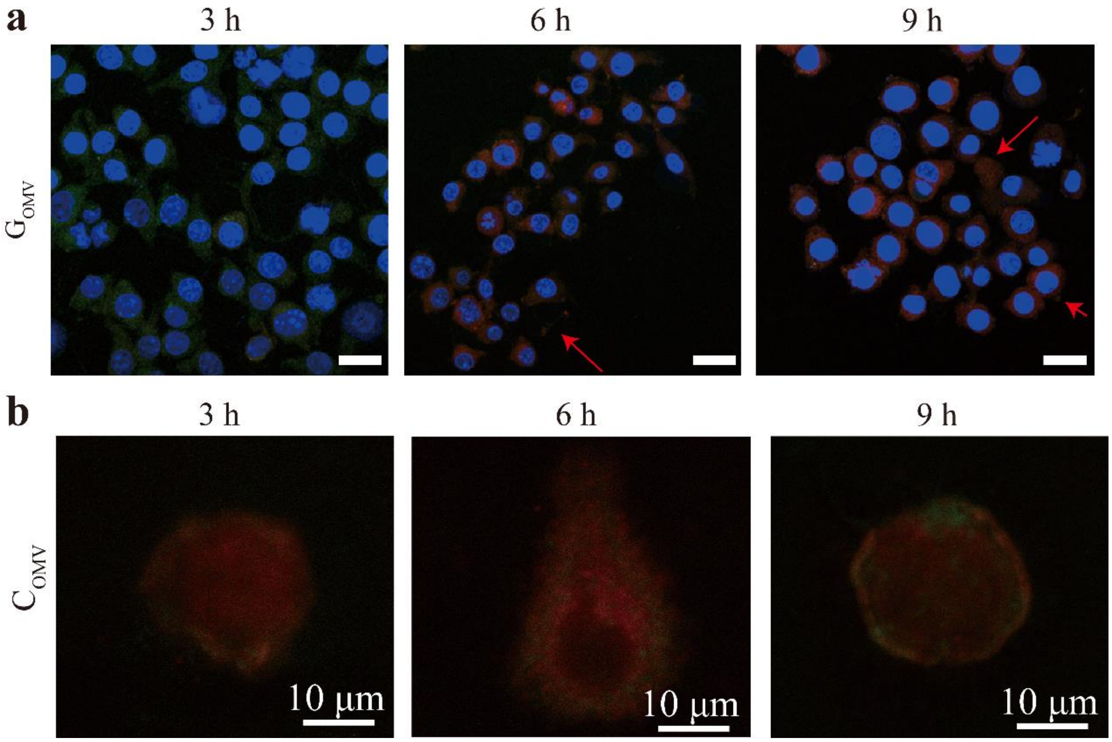

Supplementary Fig.5 Intracellular distribution of IPMN-G and IPMN-C in BMDCs. a, The intracellular distribution of G_{OMV} and IPMN-G after treatment for 3, 6 and 9 h by CLSM. Scale bar: 20 μm. b, The intracellular distribution of C_{OMV} and IPMN-C after treatment for 3, 6 and 9 h by CLSM. Scale bar: 10 μm

Figure. S6.

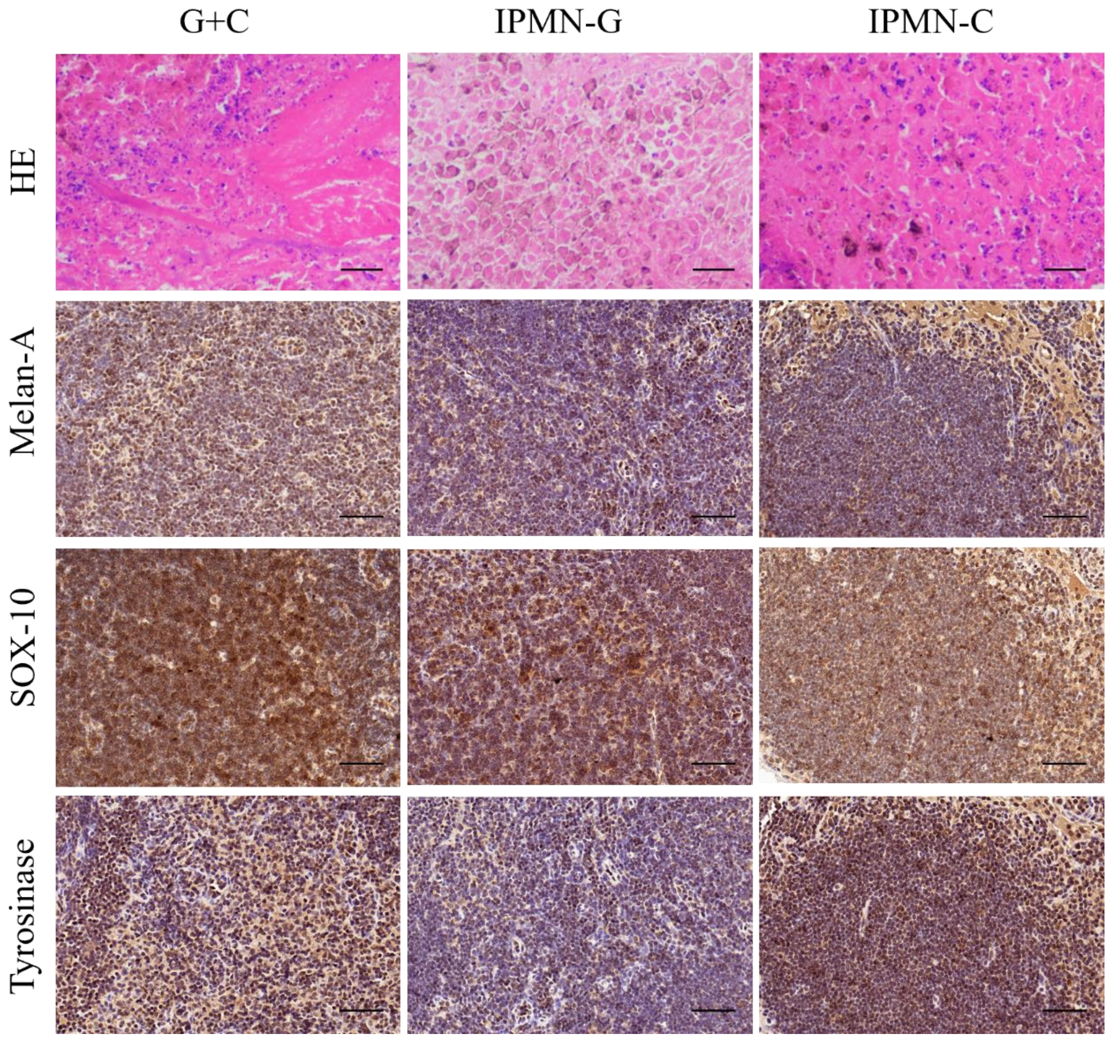

Supplementary Fig.6 H&E staining (Scale bar, 100 μm.) of tumor and histochemical analysis (Scale bar, 50 μm.) of TDLN in GC, IPMN-G, and IPMN-C groups.

Figure. S7.

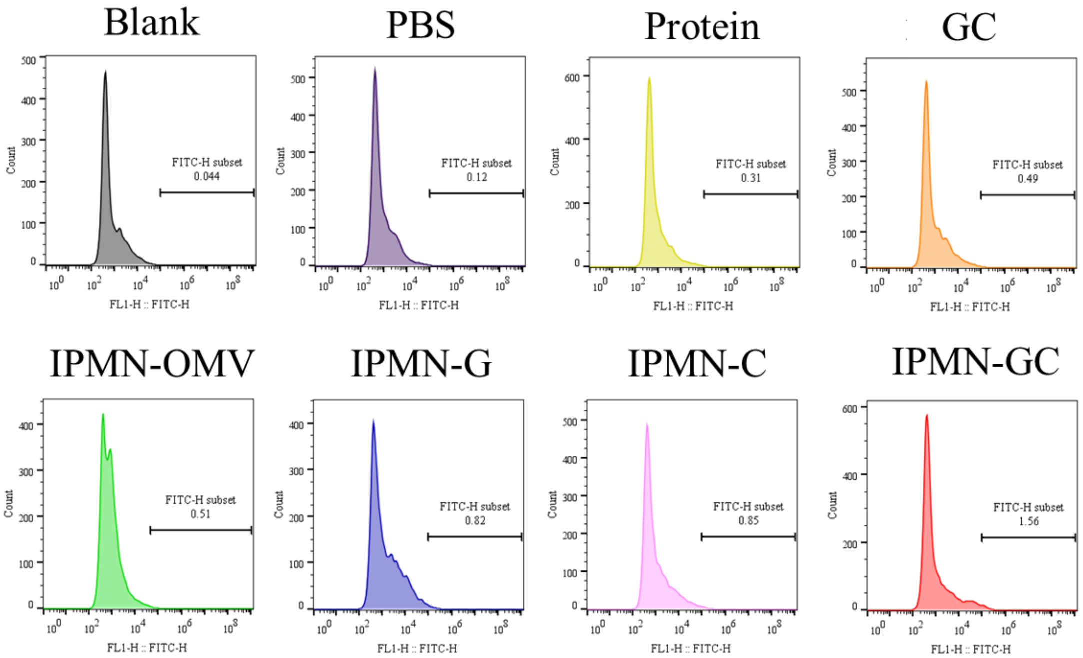

Supplementary Fig.7 DCs cell percentage in tumor site determined by FACS. (n = 3).

Figure. S8.

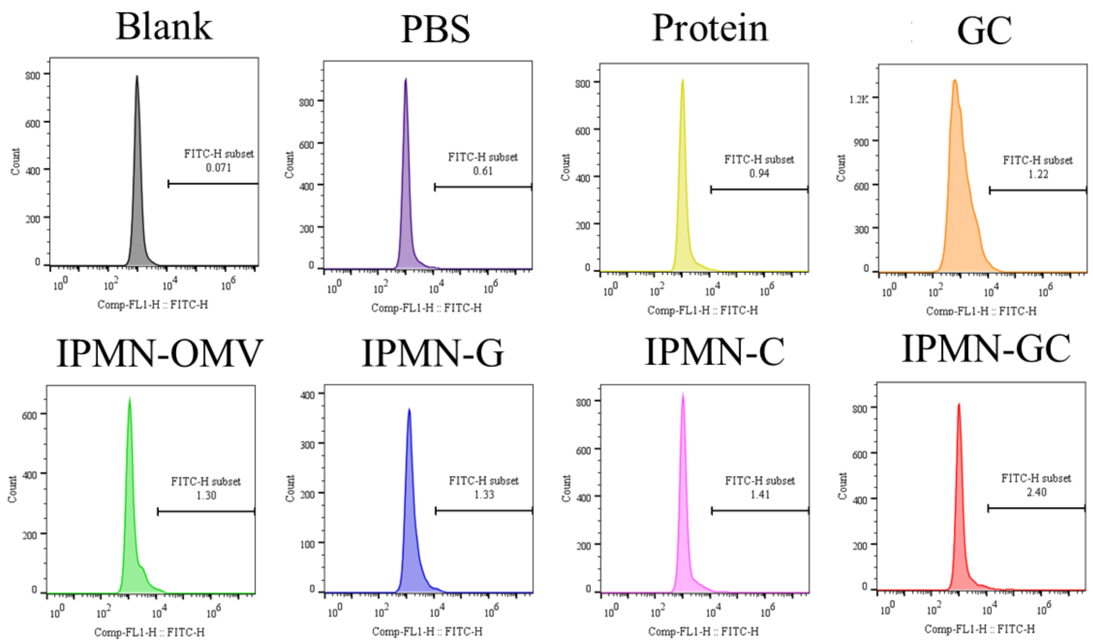

Supplementary Fig.8 DCs cell percentage in TDLN site determined by FACS. (n = 3).

Figure. S9.

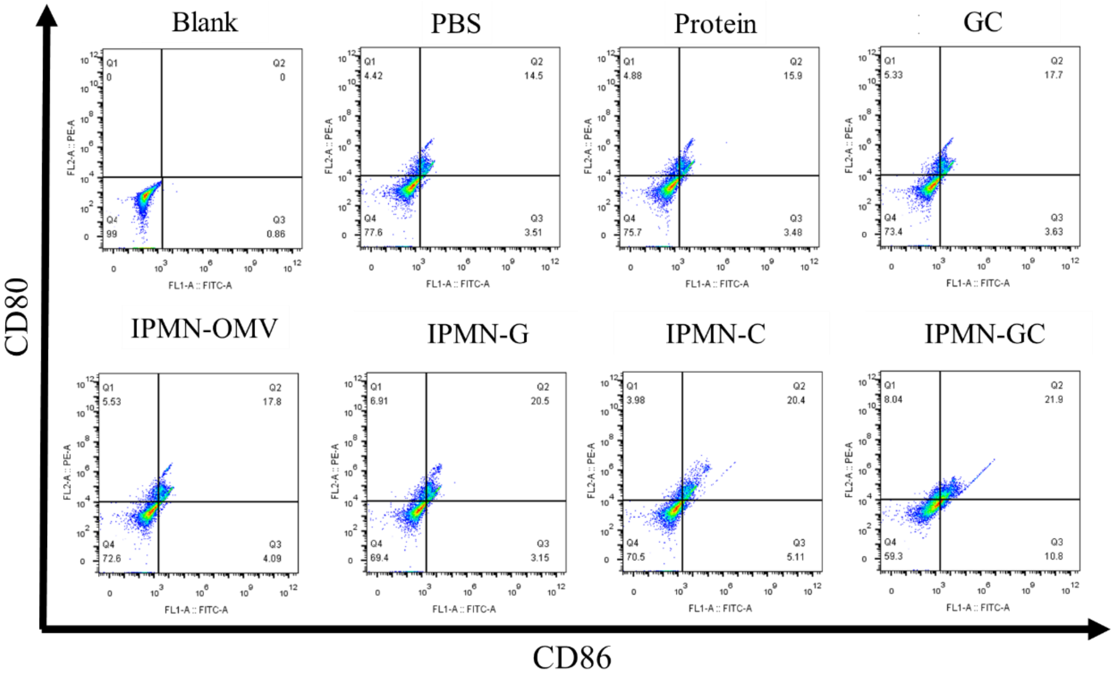

Supplementary Fig.9 The percentage of CD86+ and CD80+ cells in DC cells by FACS. (n = 3).

Figure. S10.

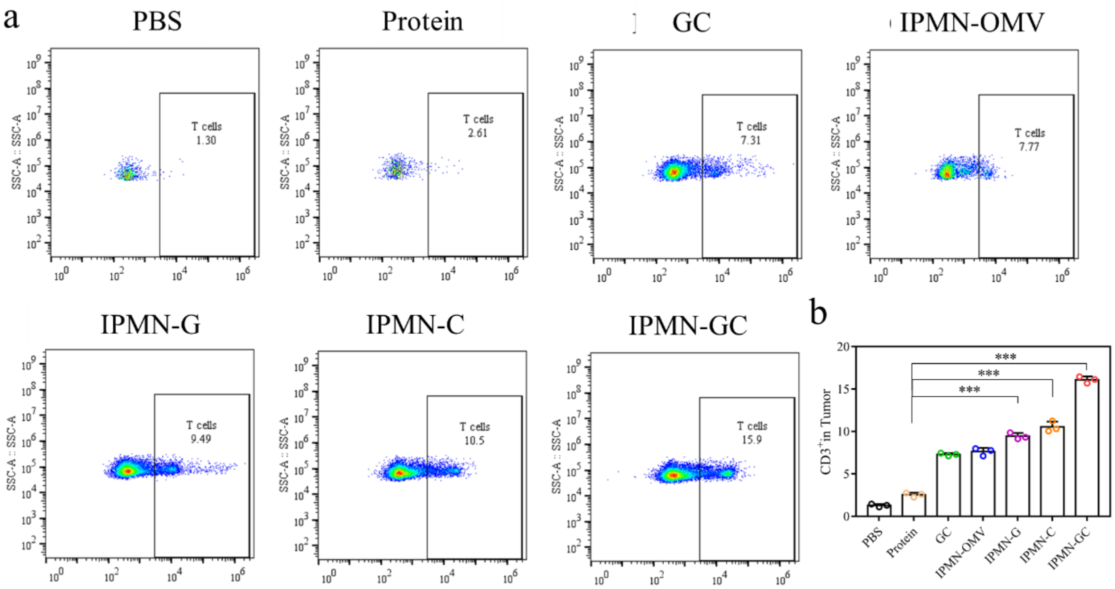

Supplementary Fig.10 Percentage of T lymphocytes at tumor sites. a, Percentage of T lymphocytes at tumor sites by FACS; b, Quantitative results of percentage of T lymphocytes at tumor sites (*p < 0.05, ***p < 0.001, n = 3).

Figure. S11.

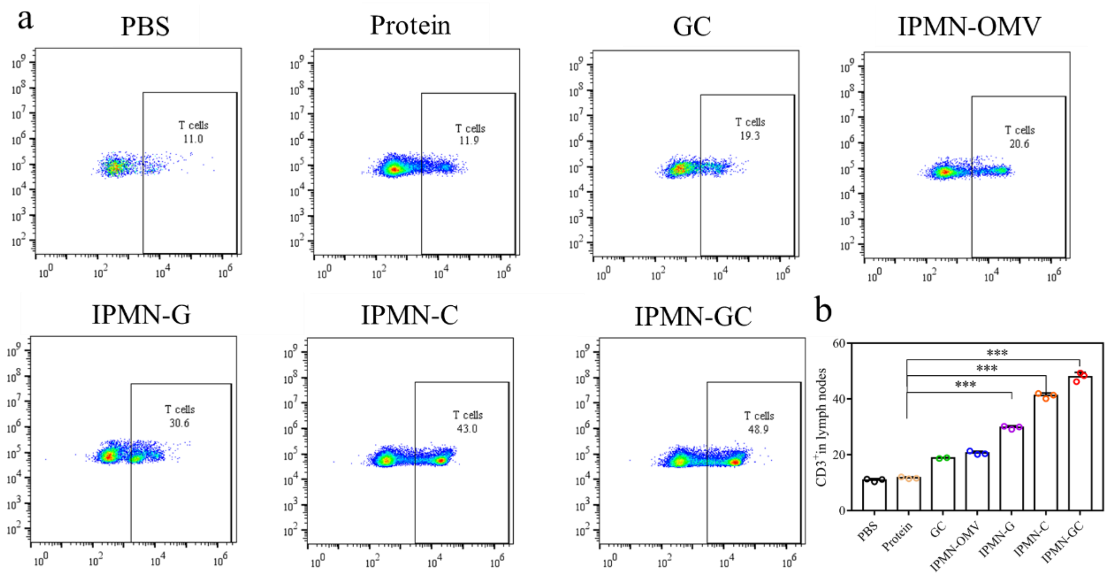

Supplementary Fig.11 Percentage of T lymphocytes in TDLN. a, Percentage of T lymphocytes in TDLN by FACS; b, Quantitative results of percentage of T lymphocytes in TDLN (**p < 0.01, ***p < 0.001, n = 3).

Figure. S12.

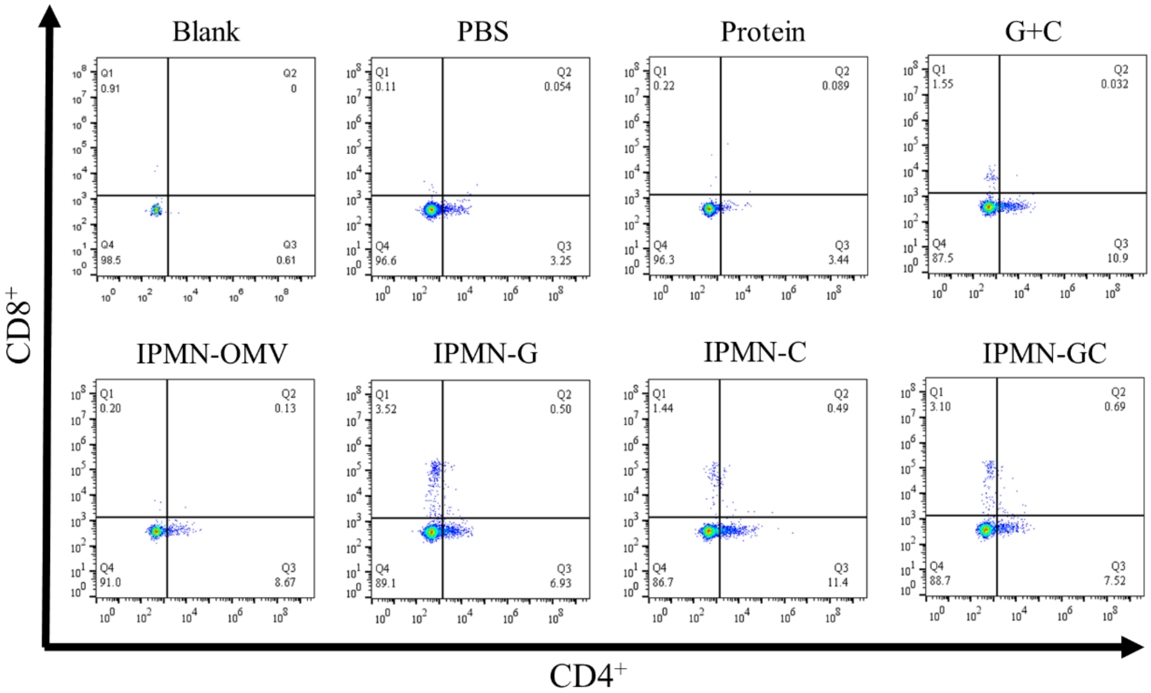

Supplementary Fig.12 The percentage of CD4+ T and CD8+ T cells in T lymphocytes at the tumor site by FACS. (n = 3)

Figure. S13.

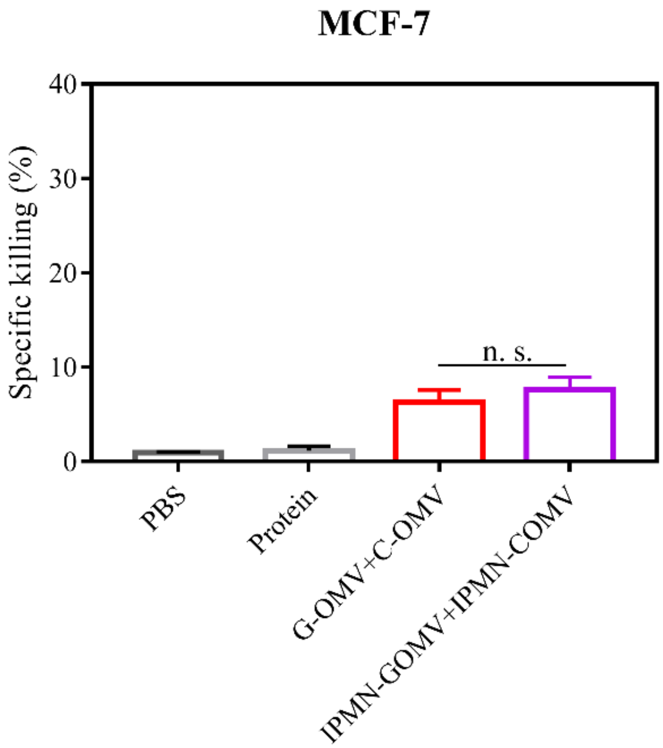

Supplementary Fig.13 The specific killing ability of splenocytes to MCF-7. (*p < 0.05, **p < 0.01, ***p < 0.001, n=6).

Figure. S14.

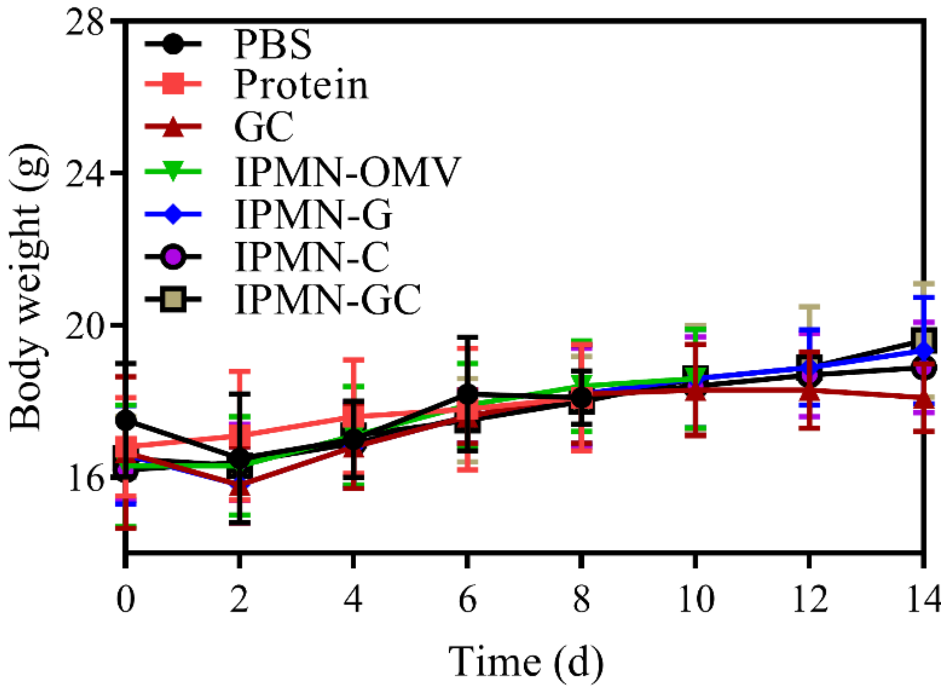

Supplementary Fig.14 The curves of tumor-bearing mice body weight (n = 6).

Figure. S15.

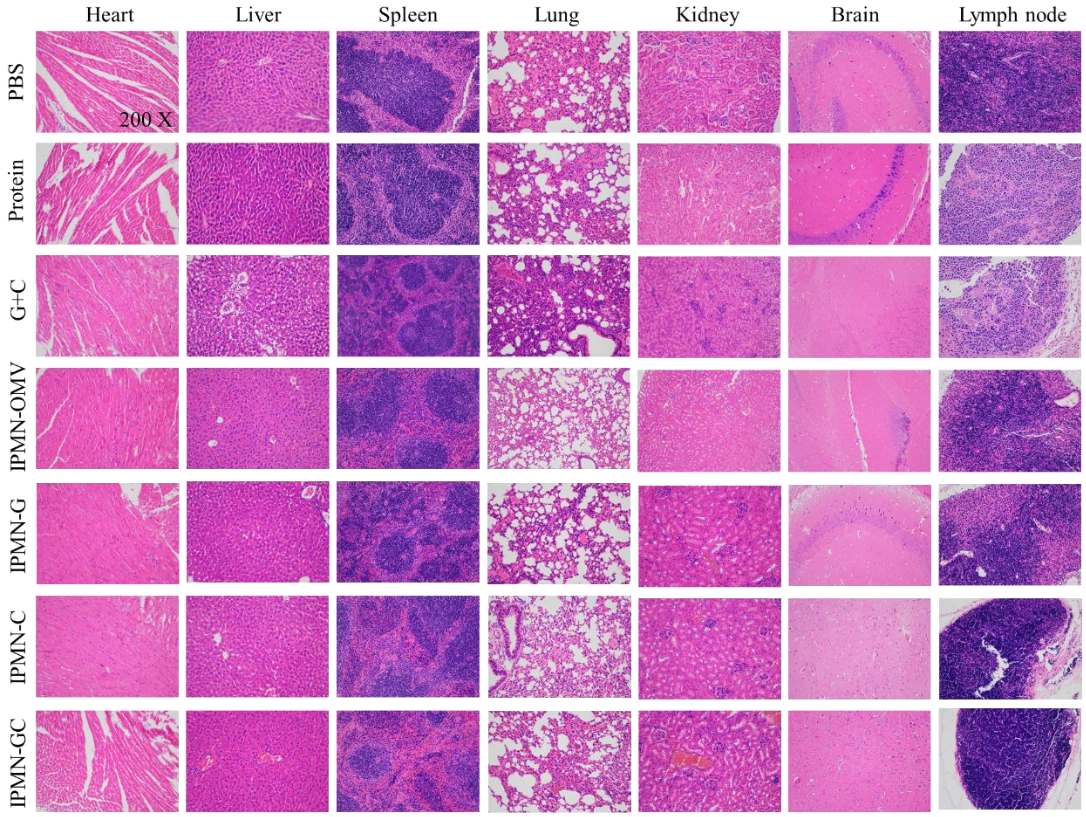

Supplementary Fig.15 H&E staining of mice organs including heart, liver, spleen, lung, kidney brain and Lymph node. (200 X)
